# Integrating animal welfare into the WHO pandemic treaty: a thematic analysis of civil society perspectives and comparison with treaty drafting

**DOI:** 10.3389/fvets.2024.1421158

**Published:** 2024-11-13

**Authors:** Ying Huang, Shisong Jiang, Nasiya Daminova, Emmanuel Kumah

**Affiliations:** ^1^School of Marxism, Yangtze Normal University, Chongqing, China; ^2^School of Law, Chongqing University, Chongqing, China; ^3^Faculty of Management and Business [Just Recovery From Covid-19? Fundamental Rights, Legitimate Governance and Lessons Learnt (JuRe) Project], Tampere University, Tampere, Finland; ^4^Department of Health Administration and Education, Faculty of Science Education, University of Education, Winneba, Ghana

**Keywords:** pandemic treaty, animal welfare, One Health, civil society, zoonotic diseases

## Abstract

The COVID-19 pandemic has exposed critical weaknesses in the global health system, highlighting the urgent need for a coordinated international approach to pandemic prevention and management. As negotiations for a new WHO pandemic treaty progress, the effective integration of animal welfare is crucial. This paper aims to investigate the perspectives of key civil society organizations on the integration of animal welfare provisions into the pandemic treaty. Through a thematic analysis of documents prepared by FOUR PAWS, Wildlife Conservation Society, and Action for Animal Health between 2020–2023, five major themes are identified: prevention of zoonotic spillover, One Health approach, animal health systems and infrastructure, sustainable and ethical animal management practices, and policy coherence and governance. A comparative analysis of these themes against the April 2024 draft of the pandemic treaty reveals areas of alignment and divergence. Due to the ongoing controversies and the need for further improvements, the WHO's intergovernmental negotiating body was unable to finalize the treaty text for the 77th World Health Assembly in May 2024, leading to an extended mandate until 2025. Based on the findings, the paper proposes recommendations to strengthen the integration of animal welfare into the treaty, arguing that incorporating these recommendations is critical for developing a transformative, equitable, and effective treaty that addresses the systemic drivers of pandemic risk.

## 1 Introduction

The COVID-19 pandemic has exposed critical weaknesses in the global health system and highlighted the urgent need for improved international cooperation and coordination in pandemic prevention, preparedness, and response (1–4). In December 2021, the World Health Assembly (WHA) agreed to establish an intergovernmental negotiating body (INB) to draft and negotiate a new international instrument on pandemics, known as the pandemic treaty (5). This treaty aims to strengthen global capacities and address the gaps exposed by COVID-19 across various aspects of pandemic management (6).

A crucial area that requires attention in the pandemic treaty is the role of animal health and welfare (7–12). There is growing recognition that human health is intricately connected to the health of animals and the environment (13, 14), with approximately 60% to 75% of new or emerging infectious diseases estimated to be zoonotic in origin, transmitted from animals to humans (15). Factors such as land use change, wildlife trade, agricultural intensification, and climate change contribute to disease spillover and amplification (16, 17). Illegal wildlife trade, in particular, poses a significant risk due to its lack of regulation, poor biosecurity measures, and the potential for direct human contact with infected animals (18, 19). However, policy and governance systems have often failed to address risks at the human-animal interface in a coordinated manner (12, 20). In this context, it is crucial to clarify two key concepts: animal welfare and pandemic prevention. Animal welfare, as defined by the World Organization for Animal Health (WOAH), refers to “the physical and mental state of an animal in relation to the conditions in which it lives and dies” (21). This holistic concept encompasses not only an animal's health but also its comfort, nourishment, ability to express innate behavior, and freedom from pain, fear, and distress (22). It is important to distinguish this from animal health, which primarily focuses on the absence of disease (23). Pandemic prevention, on the other hand, refers to proactive measures taken to reduce the risk of pathogen spillover from animals to humans and subsequent global spread (24). This includes addressing root causes such as habitat destruction, wildlife trade, and intensive animal farming practices (25). These definitions underscore the complex interplay between human activities, animal welfare, and the emergence of zoonotic diseases. The One Health approach, which recognizes the interdependence of human, animal, and environmental health (26–28), has been proposed as a unifying framework to manage these complexities and risks (29). Integrating One Health and animal welfare considerations into the pandemic treaty could help align interests, priorities, and actions across sectors to more effectively reduce pandemic threats (30, 31).

Civil society organizations (CSOs) play a vital role in advocating for the incorporation of animal welfare into global health policy and governance (32–36). CSOs working at the intersection of animal welfare, conservation, and public health possess valuable expertise, insights, and networks that can inform and shape the pandemic treaty negotiations (36, 37). Their perspectives and recommendations are essential for ensuring that the treaty adequately addresses the root causes of pandemics and promotes a One Health approach that safeguards the wellbeing of humans, animals, and ecosystems (38).

The pandemic treaty was initially planned to be adopted by the 77th WHA in May 2024. However, due to ongoing controversies and the need for further improvements in the treaty's content (39), WHO's member states decided to extend the INB's mandate to finalize the agreement by the 78th WHA in 2025 or earlier by a special session of the WHA if possible in 2024 (40). This development underscores the importance of addressing critical issues, such as the integration of animal welfare considerations, to ensure the treaty's effectiveness in preventing and managing future pandemics. On June 1, 2024, during the 77th WHA, member states agreed on a package of amendments to the International Health Regulations (IHR) (2005) to improve global preparedness, surveillance, and response to public health emergencies (41). These amendments, along with the extended timeline for the pandemic treaty, have generated momentum and urgency for integrating the insights and priorities of CSOs into the ongoing negotiations and implementation frameworks.

This paper investigates the status quo and the potential of integrating animal welfare provisions into the WHO pandemic treaty by analyzing the perspectives of key CSOs. We focus on FOUR PAWS International, Wildlife Conservation Society (WCS), and Action for Animal Health (A4AH), conducting a thematic analysis of their documents from 2020 to 2023. Our research addresses two main questions: (1) What major themes and policy priorities related to animal welfare emerge from these CSO documents? (2) How do these CSO perspectives align with or diverge from the April 2024 draft of the WHO pandemic treaty? Based on our comparative analysis, we propose recommendations to strengthen animal welfare integration in the treaty, arguing that this is crucial for developing a transformative, equitable, and effective agreement that addresses systemic drivers of pandemic risk.

The paper is structured as follows: Section 2 provides a literature review on the links between animal welfare, zoonotic diseases, and global health policy; Section 3 outlines our methodology; Section 4 presents the findings of our thematic analysis; Section 5 offers a comparative analysis of the CSOs' perspectives and the pandemic treaty draft; Section 6 puts forward recommendations; and Section 7 concludes with a discussion of the implications of our findings for the ongoing negotiations and the potential for the pandemic treaty to catalyze transformative change in global health governance. By focusing on the perspectives of CSOs and their efforts to advocate for incorporating animal welfare into the pandemic treaty, this paper contributes to the growing body of literature on the importance of integrating One Health and animal welfare considerations into global health policy and governance. Our findings highlight the critical role that CSOs can play in shaping the pandemic treaty negotiations and ensuring that the treaty adequately addresses the complex interconnections between human, animal, and environmental health. We hope that our recommendations will inform the ongoing negotiations and contribute to the development of a more comprehensive, equitable, and effective pandemic treaty.

## 2 Literature review

To provide context for our analysis, this literature review section examines the current state of knowledge on four key areas: (1) the links between animal welfare and zoonotic disease emergence (see Table 1); (2) the integration of animal welfare into global policy frameworks; (3) the development process of the proposed WHO pandemic treaty; and (4) the role of civil society in shaping global health diplomacy and treaty negotiations. By synthesizing insights from these bodies of literature, we aim to situate our research within wider academic and policy debates, and underscore the significance of understanding diverse community perspectives on upholding animal welfare within this potentially historic pandemic treaty.

**Table 1:** Synthesis of key research on animal welfare and zoonotic spillover.

**Research focus**	**Study**	**Species/ecosystems involved**	**Geographic scope**	**Key findings**
Zoonotic disease prevalence and trends	Taylor et al. (42)	Multiple species	Global	61% of human pathogens are zoonotic; 75% of emerging pathogens are zoonotic
	Jones et al. (43)	Multiple species	Global	60.3% of EIDs are zoonoses; 71.8% originate in wildlife
	Smith et al. (44)	Human populations	Global	44% increase in outbreaks and 25% increase in unique diseases between 1980s and 2010-2013
Environmental and ecological factors	Allen et al. (49)	Multiple wildlife species	Global	Zoonotic EID risk elevated in forested tropical regions with high biodiversity
	Murray et al. (50)	Multiple species	Global	Mammalian biodiversity is the strongest predictor of infectious disease co-occurrence
	Guernier et al. (51)	Multiple species	Global	Negative relationship between latitude and parasite species richness
	Murray and Daszak (52)	Multiple species	Global	Two hypotheses for mechanisms of land-use change leading to viral emergence
	Perfecto et al. (53)	Agricultural ecosystems	Global	Diverse agroecological matrices may decrease probability of zoonosis emergence
	Carlson et al. (54)	Multiple species	Global	Projected 15,000 new cross-species viral transmissions by 2070 due to climate change
	Fisher et al. (55)	Multiple species	Global	Fungal diseases pose increasing threat to animal, plant, and ecosystem health
Wildlife trade and urbanization	Scheffers et al. (56)	5,579 mammal species	Global	Estimated scale of global wildlife trade and its potential impact on disease dynamics
	Shivaprakash et al. (57)	Mammals in wildlife trade	Global	26.5% of traded mammals harbor 75% of known zoonotic viruses
	Greatorex et al. (59)	Multiple mammal species	Lao PDR	1,937 mammals from 12 taxonomic families observed for sale, capable of hosting 36 zoonotic pathogens
	Aguirre et al. (58)	Multiple species	Global	Illegal trade exacerbates zoonotic spillover risks due to lack of regulation and poor conditions
	Rush et al. (18)	Multiple species	Global	Over 60% of pathogens in illegal wildlife trade have known zoonotic potential
	Firth et al. (60)	Norway rats	New York City, USA	Urban rats harbor a wide range of known and novel viruses, including zoonotic pathogens
	Bradley and Altizer (61)	Urban wildlife	Global	Urbanization can increase transmission among urban-adapted hosts
	Hassell et al. (62)	Multiple species	Urban areas	Urbanization creates diverse wildlife-livestock-human interfaces, increasing zoonotic risk
Agricultural practices and zoonoses	Graham et al. (63)	Livestock	Global	High stocking densities and antimicrobial use create conditions for pathogen amplification
	Dhingra et al. (64)	Poultry	Global	39 HPAI emergence events, mostly in commercial poultry systems in high-income countries
	Jones et al. (65)	Wildlife, livestock	Global	Agricultural intensification associated with increased risk of zoonotic disease emergence
	Hollenbeck (66)	Livestock	Global	CAFOs contribute to the emergence of infectious diseases
	Hayek (67)	Livestock	Global	Intensive animal agriculture creates a “trap” of zoonotic disease risks
Socioeconomic and human behavioral factors	Pedersen and Davies (68)	Primates	Africa, Amazonia	Central Africa and Amazonia are hotspots for cross-species transmission events
	Beirne (69)	Wildlife	Global	Role of wildlife trade in pathogen spillover from criminological perspective
	Astbury et al. (70)	Multiple species	Global	Review of public policies targeting determinants of zoonotic spillover
	Stephen et al. (71)	N/A	Global	Identified implementation gap in emerging disease risk management strategies
	Cocco et al. (72)	Dogs	Italy	Correlation between animal welfare in shelters and antimicrobial resistance
Pathogen characteristics and host-pathogen interactions	Smith and Wang (76)	Bats	Global	Bats are reservoirs for various zoonotic viruses, including SARS, Nipah, and Ebola
	Li et al. (78)	Bats	China	Bats identified as natural reservoirs of SARS-like coronaviruses
	Ge et al. (77)	Bats	China	Isolation of SARS-like coronavirus from bats that can use human ACE2 receptor
	Olival and Hayman (79)	Bats	Global	Bats are important reservoirs for filoviruses, including Ebola and Marburg viruses
	Han et al. (80)	Rodents	Global	Predicted hotspots of novel rodent reservoir diversity in Middle East, Central Asia, and Midwestern US
	Luis et al. (81)	Bats and rodents	Global	Comparison of bats and rodents as reservoirs of zoonotic viruses
	Pernet et al. (75)	Bats, humans	Cameroon	Evidence of henipavirus spillover into human populations in Africa
	Wardeh et al. (73)	Multiple species	Global	Comprehensive database of host-pathogen interactions and global distributions
	Johnson et al. (74)	Multiple species	Global	Viruses with high host plasticity more likely to cause pandemics
	Han et al. (80)	Mammals	Global	Review of zoonotic potential in different mammalian groups
	Guo et al. (215)	Bats, rodents, shrews	China	Hantaviruses might have first appeared in bats or shrews before emerging in rodents
	Negredo et al. (216)	Bats	Spain	Discovery of a novel ebolavirus-like filovirus in European bats
	Cui et al. (217)	Bats, coronaviruses	Asia	Host shifts have occurred in recent evolutionary history of bat coronaviruses
Conceptual frameworks, models, and one health approaches	Morse et al. (12)	Multiple species	Global	Need for improved surveillance and pre-emptive approaches to pandemic prevention
	Plowright et al. (46)	Multiple species	Global	Synthetic framework for understanding animal-to-human transmission
	Lloyd-Smith et al. (86)	Multiple species	Global	Importance of modeling zoonoses across multiple host species and disciplines
	Johnson et al. (74)	Multiple species	Global	Application of community ecology principles to infectious disease research
	Ellwanger and Chies (48)	Multiple species	Global	Overview of factors involved in zoonotic spillover events
	Karesh et al. (82)	Multiple species	Global	>60% of human infectious diseases caused by pathogens shared with animals
	Kelly et al. (218)	Multiple species	Global	PREDICT project: 90 publications improving understanding of zoonoses and emergence factors
	Olival et al. (219)	Mammals	Global	Total number of viruses per host species and proportion of zoonotic viruses are predictable
	Thumbi et al. (214)	Livestock, humans	Western Kenya	Incidence of human illness increased 1.31-fold for every 10 cases of animal illness or death
	Tarazona et al. (91)	Multiple species	Global	Importance of One Health for animal welfare and human-animal relationships
	Pinillos et al. (220)	Multiple species	Global	One Welfare framework linking animal welfare, human wellbeing, and environment
	Düpjan and Dawkins (221)	Multiple species	Global	Environments promoting animal well-being can reduce disease susceptibility
	Alders and Rushton (83)	Livestock	Global	Discussion on limits of animal production systems for tolerable animal welfare and disease risks
	Stel et al. (84)	Livestock	Global	Strategies for mitigating zoonotic risks in intensive farming
	Overgaauw et al. (222)	Companion animals	Global	One Health perspective on human-companion animal relationship and zoonoses
	Radeski et al. (223)	Multiple species	Macedonia	Evaluation of Animal Welfare Center from One Health perspective
	Liguori et al. (93)	Multiple species	Global	One Health challenges in animal-assisted interventions
	Maes et al. (94)	Pigs	Global	Critical reflection on intensive pork production and animal welfare
	Gongal and Ofrin (95)	Multiple species	Asia-Pacific	One Health approach in emergency preparedness and response
	Goetschel (96)	Multiple species	Global	Proposal for UN Convention on Animal Health and Protection
	Berezowski et al. (97)	Multiple species	Europe	Prioritization of zoonotic diseases for One Health surveillance
	Koralesky et al. (90)	Multiple species	Global	One Welfare in animal sheltering and protection
	Warwick and Steedman (92)	Multiple species	Global	Wildlife-pet markets in a One Health context
	Mota-Rojas et al. (88)	Multiple species	Global	Animal abuse as indicator of domestic violence: One Health, One Welfare approach
	Plowright et al. (85)	Multiple species	Global	Importance of maintaining ecological conditions to reduce pathogen spillover risk
	Markotter et al. (47)	Multiple species	Global	Shift from response to reducing risk at the source for zoonotic spillover prevention

### 2.1 Intersections of animal welfare and zoonotic disease emergence

#### 2.1.1 Zoonotic disease prevalence and trends

Recent decades have seen a growing body of evidence that documents the intricate connections between animal welfare and human health risks. They reveal a complex web of interactions that contribute to the emergence and spread of zoonotic diseases. A study by Taylor et al. (42) laid the groundwork for understanding these relationships by analyzing 1,415 species of human pathogens, with striking findings showing that 61% (868 species) of these pathogens were zoonotic capable of transmission between humans and animals. Even more significantly, 75% of emerging pathogens were zoonotic, highlighting the critical role of animal reservoirs in human disease emergence. Building on such foundation, Jones et al. (43) conducted a comprehensive analysis of 335 emerging infectious disease (EID) events between 1940 and 2004, indicating that 60.3% of EIDs were zoonoses, with 71.8% of these originating in wildlife. Besides, they also noted a significant increase in EID events over time, even after controlling for reporting bias. This trend underscores the growing importance of understanding and mitigating zoonotic risks, particularly in the context of rapidly changing global environments and human-animal interactions. Further emphasizing this trend, Smith et al. (44) analyzed a 33-year dataset (1980–2013) of 12,102 outbreaks of 215 human infectious diseases, observing a 44% increase in the number of outbreaks and a 25% increase in the number of unique diseases between the 1980s and 2010–2013. In the context of the extraordinary situation caused by the COVID-19 pandemic, the IPBES (The Intergovernmental Science-Policy Platform on Biodiversity and Ecosystem Services) Workshop Report on Biodiversity and Pandemics, bringing together over 20 experts, highlighted how increasing contacts between humans, livestock, and wildlife, driven by food demand, land-use change, wildlife trade and trafficking, climate change impacts, and other anthropogenic factors are escalating the likelihood of zoonotic spillovers (45). It sheds light again [for others, see Plowright et al. (46), Markotter et al. (47), and Ellwanger and Chies (48)] on the critical relationship between biodiversity loss and ecosystem degradation, undermining wildlife population health and increasing opportunities for pathogen transmission between species (45).

#### 2.1.2 Environmental and ecological factors

Indeed, the role of environmental and ecological factors in zoonotic disease emergence has been a focus of recent research. Allen et al. (49) used boosted regression tree models to analyze the demographic, environmental, and biological correlates of disease emergence. Their findings indicated that zoonotic EID risk is elevated in forested tropical regions experiencing land-use changes and where wildlife biodiversity (mammal species richness) is high, providing a new global hotspot map of spatial variation in zoonotic EID risk. Murray et al. (50) analyzed the global occurrence patterns of 187 human infectious diseases across 225 countries, finding that mammalian biodiversity was the strongest predictor of infectious disease co-occurrence overall. This finding challenges simplistic notions about biodiversity loss and disease emergence. Guernier et al. (51) further contributed to this understanding by demonstrating a significant negative relationship between latitude and parasite species richness, suggesting that tropical regions may be hotspots for disease diversity. In addition, the impact of land-use change on viral emergence was explored by Murray and Daszak (52), who proposed the “perturbation” hypothesis and the “pathogen pool” hypothesis to provide a framework for understanding how human activities can create conditions conducive to zoonotic spillover events. On the relationship between agriculture and zoonoses, Perfecto et al. (53) suggested that diverse agroecological matrices may decrease the probability of zoonosis emergence. Also, climate change has been increasingly recognized as a critical driver of zoonotic disease risk. For instance, Carlson et al. (54) used a phylogeographical model of the mammal-virus network and projections of geographical range shifts for 3,139 mammal species to simulate potential hotspots of future viral sharing under climate-change and land-use scenarios for the year 2070. Their study predicts that species will aggregate in new combinations at high elevations, in biodiversity hotspots, and in areas of high human population density in Asia and Africa, causing the cross-species transmission of their associated viruses an estimated 4,000 times. Interestingly, Fisher et al. (55) broadened the scope of environmental threats by exploring the role of fungal pathogens in animal, plant, and ecosystem health, arguing that nascent fungal infections will cause increasing attrition of biodiversity unless steps are taken to tighten biosecurity worldwide.

#### 2.1.3 Wildlife trade and urbanization

In addition to environmental and ecological factors, wildlife trade and urbanization have emerged as critical factors in zoonotic disease emergence as well. For example, Scheffers et al. (56) estimated that 24% (7,638 species) of over 31,500 terrestrial bird, mammal, amphibian, and squamate reptile species are traded globally, providing a stark illustration of the scale of the wildlife trade and its potential impact on biodiversity and disease dynamics. Furthermore, Shivaprakash et al. (57) found that 26.5% of mammals in the wildlife trade harbor 75% of known zoonotic viruses, a level much higher than that found in domesticated and non-traded mammals. However, it is noteworthy that the illegal wildlife trade poses an even greater threat due to a lack of regulation and poor hygiene conditions. Aguirre et al. (58) highlighted how illegal wildlife trade can exacerbate the risks of zoonotic spillover, emphasizing the need for a transdisciplinary approach to mitigate these risks. A study by Greatorex et al. (59) in Lao PDR found that during 21 observational surveys at seven markets, 1,937 alive or fresh dead mammals were observed for sale, including mammals from 12 taxonomic families previously documented to be capable of hosting 36 zoonotic pathogens. Rush et al. (18) further emphasized that over 60% of pathogens in illegal wildlife trade have known zoonotic potential. Regarding the crucial role played by urban environments in zoonotic disease dynamics, Firth et al. (60) conducted a comprehensive survey of Norway rats in New York City and identified a wide range of known and novel viruses. Bradley and Altizer (61) and Hassell et al. (62) explored how urbanization influences wildlife-pathogen interactions, noting that while urbanization can reduce the abundance of some wildlife parasites, it can also increase transmission among urban-adapted hosts.

#### 2.1.4 Agricultural practices and zoonoses

Besides, existing literature has also identified agricultural practices, particularly intensive animal farming, as significant contributors to zoonotic disease risk. Graham et al.' (63) analysis of the animal-human interface in industrial food animal production is an example of this kind, especially in the sense of outlining key transmission dynamics within industrialized livestock production systems. Another example refers to Dhingra et al.'s (64) data analysis of the highly pathogenic avian influenza (HPAI) H5 and H7 virus emergences in poultry, which identify a total of 39 independent H7 and H5 LPAI to HPAI conversion events, mostly in commercial poultry systems in high-income countries. Moreover, Jones et al. (65) found that agricultural intensification was associated with an increased risk of zoonotic disease emergence. Hollenbeck (66) specifically examined the role of Concentrated Animal Feeding Operations (CAFOs) in the emergence of infectious diseases. Hayek (67) argued that intensive animal agriculture creates a “trap” of zoonotic disease risks, suggesting that preventing zoonotic diseases requires international coordination to reduce the high demand for animal-sourced foods and improve forest conservation governance.

#### 2.1.5 Socioeconomic and human behavioral factors

Several researchers have paid attention to uncovering the importance of socioeconomic factors in zoonotic disease emergence. Pedersen and Davies (68) explored the risk of disease transfer between wild primates and humans, highlighting central Africa and Amazonia as hotspots for cross-species transmission events. Their work underscores the need to consider both biological and social factors in predicting and preventing zoonotic disease emergence. Differently, Beirne (69) examined the role of wildlife trade in pathogen spillover and advocated for the abolition of all wildlife trade and the reclamation of wildlife habitat through a criminological perspective. Astbury et al. (70) reviewed public policies targeting determinants of zoonotic spillover, highlighting the need for evidence-based interventions. By identifying the implementation gap in emerging disease risk management, Stephen et al. (71) accentuated the need for more research on the effectiveness, acceptability, and sustainability of risk reduction interventions in real-world contexts. Lastly, Cocco et al.'s work demonstrated the interconnectedness of public health issues and animal welfare by attending to the correlation between animal welfare in shelters and antimicrobial resistance (72).

#### 2.1.6 Pathogen characteristics and host-pathogen interactions

The complex nature of zoonotic disease emergence and the multiple factors around pathogen characteristics and host-pathogen interactions (73, 74) have been explored from various angles in the existing literature. In particular, studies have examined the role of specific animal groups in zoonotic disease transmission, the evolutionary relationships between pathogens and their hosts, and the discovery of novel pathogens with zoonotic potential [such as henipavirus spillover, see Pernet et al. (75)]. In light of this, bats have been identified as important reservoirs for numerous zoonotic viruses. Smith and Wang (76) provided a solid review of the public health implications of bat-derived zoonotic viral disease outbreaks. The discovery of SARS-like coronaviruses in bats (77, 78) further underscored the importance of these animals in the ecology of emerging zoonoses. Olival and Hayman (79) reviewed the current knowledge and future directions for filoviruses in bats; and they disclosed the necessity for a more unified, global surveillance strategy. As with the scholarly interests in bats, the role of rodents in zoonotic disease transmission has also been extensively studied. For instance, Han et al. (80) applied machine learning techniques to predict rodent species with a high probability of harboring undiscovered zoonotic pathogens. Luis et al. (81) compared bats and rodents as reservoirs of zoonotic viruses in order to offer insights into the unique characteristics of these mammalian orders in disease transmission.

#### 2.1.7 Conceptual frameworks, models, and One Health approaches

As our understanding of these complex relationships continues to evolve, there is a growing call for a shift from reactive to pre-emptive approaches in global pandemic strategy in the existing literature [among others, see Karesh et al. (82), Alder sand Rushton (83), Stel et al. (84), and Plowright et al. (85)]. In particular, Morse et al. (15) emphasized the need for improved surveillance, diagnostic capabilities, and risk assessment approaches to identify microbes most likely to cause human disease before they emerge as significant threats. Plowright et al. (46) proposed a synthetic framework for animal-to-human transmission that integrates relevant mechanisms (in other words, a transdisciplinary investigation of spillover events) and discovered that all zoonotic pathogens must overcome a hierarchical series of barriers to cause spillover infections in humans (43). Lloyd-Smith et al. (86) emphasized the need for a new generation of models that address a broader set of pathogen life histories and integrate across host species and scientific disciplines. Further, Johnson et al. (17) elaborated on how infectious disease research can benefit from applying principles from community ecology, from which new analytical tools can be developed to understand complex multi-host, multi-pathogen systems. More importantly, in response to these multifaceted challenges in question, the concept of One Health has gained significant traction in recent years and has further expanded to include One Welfare, which caters to the links between animal welfare and health, human wellbeing, and environmental health (87–91). In the growing amount of literature, the concept has been applied to various contexts, including animal sheltering and protection (90), wildlife-pet markets (92), and the relationship between animal abuse and domestic violence (88). It has also been extended to areas such as animal-assisted interventions (93), intensive pork production (94), and emergency preparedness (95). Given its significance, therefore, researchers have also explored the integration of One Health principles into policy and practice. Proposing a UN Convention on Animal Health and Protection to further the enforceability of a One Health approach is a scholarly effort of such kind at the international level (95–97).

Despite the increasing recognition of One Health principles, it is vital to keep in mind that an anthropocentric bias persists in zoonotic disease research, policy, and practice. This human-centric approach undermines efforts to address complex health challenges holistically (98, 99). From animal culling practices (100) to antimicrobial resistance strategies (101), human interests often overshadow animal welfare and environmental concerns. Even pivotal moments like the COVID-19 pandemic have failed to significantly shift this paradigm (102). The instrumentalization of animals in One Health approaches (103) and the narrow focus on human outcomes in health metrics (98) reflect deeply ingrained anthropocentric values. This bias limits the effectiveness of zoonotic disease prevention and management strategies (104) and overlooks crucial interconnections between human and more-than-human worlds (105). Thus, addressing this anthropocentric bias is essential for developing truly integrated, ethical, and effective approaches to global health challenges (106).

### 2.2 Animal welfare in global policy frameworks

The importance of considering animal health and welfare in global policymaking has also been increasingly recognized (107, 108). The WOAH, FAO, and WHO have a long history of collaboration, formalizing a shared One Health approach in 2010 that emphasizes the interconnectedness of human, animal, and environmental health (109). While not explicitly mentioned within the targets, animal welfare is an essential consideration for achieving the 2030 Agenda for Sustainable Development (110). Good animal welfare practices are deeply connected to the environmentally sound management of chemicals and waste (SDG 12.4.1) and contribute to responsible consumption and production patterns (SDG 12) (8, 111). WHO, FAO, and World Bank have also worked to include animal welfare in country-level One Health investment frameworks and highlight links with antimicrobial resistance risks (112).

However, critics have argued that policy traction has been inadequate with continued “siloed” governance across human health, livestock, and wildlife sectors enabling externalization of animal welfare and environmental costs (8). There remain gaps in terms of high-level political mandates, coordinated regulations, and practical guidance (113). The lack of systemic and integrated changes also diminishes pandemic preparedness (114). As Bonilla-Aldana et al. point out, the COVID-19 pandemic has highlighted the need for a more integrated One Health approach that considers the interconnectedness of human, animal, and environmental health to better prevent and respond to zoonotic disease outbreaks (115).

In summary, a growing evidence base supports animal welfare as an intrinsic component of pandemic preparedness strategies (7). However, political and regulatory obstacles have hindered progress in this area (116). Addressing the challenges at the human-animal-environment interface requires a multidisciplinary approach and changes in attitudes, policies, and resource allocation (117). The proposed pandemic treaty represents a significant political opportunity and a high-level commitment from governments to enhance global pandemic preparedness and response (118). The integration of animal welfare considerations into the treaty could have substantial impacts by mandating changes across sectors.

### 2.3 Pandemic treaty development process

On December 1, 2021, the WHA adopted a landmark consensus resolution co-sponsored by various countries to kickstart a global process for developing an international instrument on pandemic prevention, preparedness, and response (5). This built on earlier recommendations made by various independent panels assessing global failures that enabled COVID-19 to turn into a devastating humanitarian crisis. These high-level panels, like the Independent Panel for Pandemic Preparedness and Response and the G20 High-Level Independent Panel, had strongly urged major reforms to the global health architecture and universal, binding commitments from governments to be better equipped for the next pandemic (119, 120).

The ambitious resolution decided to establish an intergovernmental negotiating body (INB), open to all WHA members, with the mandate to draft and negotiate an agreement on pandemics over the next 2 years (5). The INB initially aimed to submit its final outcome to the 77th WHA in May 2024 (121). However, due to disagreements among member states on some provisions during the INB negotiation process, the INB was unable to finalize the pandemic treaty proposal in time for the 77th WHA. Even if so, the UN Secretary-General endorsed these efforts, highlighting that “the world must improve surveillance of viruses, strengthen health systems, and make the promise of Universal Health Coverage a reality. We must renounce the moral and medical disaster of rich countries hoarding and controlling pandemic healthcare supplies, and ensure everyone has access to diagnostics, treatments and vaccines. And we must strengthen the World Health Organization's authority and financing” (122).

The INB process, which began in February 2022, has already included nine rounds of meetings as of March 1, 2024. These meetings have brought together government representatives and affected communities to share views, along with expert symposia, open consultations, and the publication of discussion papers on the potential scope and content of the pandemic instrument. The INB Bureau has successively issued the Conceptual zero draft (123), Zero draft (124), Bureau's text (125), Proposal for negotiating text (126), Revised draft of the negotiating text of the WHO Pandemic Agreement (127), and Proposal for the WHO Pandemic Agreement (128) integrating government and stakeholder inputs received. Multi-stakeholder hearings have enabled accredited non-state participants to provide comments and written submissions (129). In 2023, the INB decided to organize its work in collaboration with the Working Group on Amendments to the International Health Regulations (2005) (WGIHR) to ensure coherence and reduce duplication between the two processes (130). The INB and WGIHR held joint plenary sessions to discuss issues such as the “Public health alert—public health emergency of international concern—pandemic continuum” and “Pandemic prevention, preparedness, surveillance, and One Health.”

In the INB meetings, key issues under discussion include governance and oversight mechanisms (131–135), benefit-sharing arrangements around access to medical countermeasures and pathogens (136–138), strengthening preparedness and response capacities (139), financing (140–142), equity (143–146), and assistance to developing countries (143, 147, 148). The INB has also addressed the participation of relevant stakeholders in its work and has agreed to include additional stakeholders in its deliberations (149). However, the prevention of pandemics, identified as a core objective within the initial WHA resolution, has not yet been extensively translated into INB outcomes or draft provisions, despite some increased attention in later meetings (150, 151). This is despite widespread expert and scientific consensus that tackling anthropogenic drivers at the source would be significantly more effective and economical for reducing pandemic risks compared to reacting after spillover or emergence, as the world has done with COVID-19 (152, 153). According to a thematic analysis of member state interventions at the INB sessions in July 2022, a key concern is that the early drafts of the pandemic treaty may prioritize reactive measures over the proactive prevention of spillover events, failing to fully address the ecological roots of pandemics (154). While there has been some attention toward One Health and the human-animal-environment interface in the INB's work (10), civil society organizations argue that these aspects need further strengthening and operationalization to align with the WHO's mandate.

On June 1, 2024, during the 77th WHA, member states decided to extend the mandate of the INB to finish its work on negotiating the pandemic treaty within a year, by the WHA in 2025, or earlier if possible at a special session of the WHA in 2024 (40). In the same session, the WHA agreed on a package of critical amendments to the IHR (2005) to improve global preparedness, surveillance, and response to public health emergencies, including pandemics (41). The key amendments include introducing a definition of a pandemic emergency to trigger more effective international collaboration; a commitment to solidarity and equity on strengthening access to medical products and financing; establishment of the States Parties Committee to facilitate the effective implementation of the amended Regulations; creation of National IHR Authorities to improve coordination of implementation within and among countries (41). INB Co-Chairs Precious Matsoso and Roland Driece highlighted the consensus among member states on the need for a further instrument to help the world better fight a full-blown pandemic and the momentum provided by the IHR amendments to finalize the pandemic agreement. Matsoso stated, “[t]here was a clear consensus amongst all Member States on the need for a further instrument to help the world better fight a full-blown pandemic,” while Driece added, “[t]oday's great result in approving amendments to the International Health Regulations will provide the momentum we need to finalize the Pandemic Agreement” (155).

### 2.4 Civil society in global health diplomacy and treaty negotiations

Global health diplomacy, according to Kickbusch et al. for instance, can be perceived as political change grounded in the belief that negotiation is the most effective, ethical, and sustainable way of promoting better health for all where state and non-state entities leverage coordinated influence (156). Within academic literature and policy practice, there has been increasing recognition over the past few decades of the importance of engaging CSOs and non-state stakeholders as partners alongside governments and international institutions like WHO in these negotiations toward health policy decisions (31, 157–159). This recognition stems from accumulating evidence that CSO participation, if conducted meaningfully, can enhance transparency and democratic legitimacy, lend issue expertise, facilitate implementation of global decisions locally, mobilize resources or political constituencies, and improve health outcomes (33, 160, 161). For instance, in their analysis of the role of non-state actors in advancing health diplomacy, scholars have argued that CSOs can play a critical role in providing expertise, mobilizing resources, and holding governments accountable (37). Similarly, researchers have suggested that civil society participation in global public-private partnerships for health can enhance transparency, accountability, and legitimacy, as well as contribute to better health outcomes (36). These perspectives highlight the value proposition of civil society engagement in strengthening democratic global health policymaking and ensuring that governments adhere to their political agreements and health commitments.

In this regard, CSOs have played pivotal roles in shaping agendas and inserting language into various landmark global health agreements (32, 34, 35, 162). For instance, as part of a global alliance of non-governmental organizations and activist networks, civil society was credited as crucial during the final phases of the WHO Framework Convention on Tobacco Control negotiations to raise political attention onto tobacco-related issues, counter industry lobbying, secure key victories around recognition of tobacco's severe public harms, protection of public health policies, demand-reduction strategies and use of tobacco litigation, and enable unanimous adoption (163, 164) despite initial reluctance of several high-income governments (165). Besides, the establishment of a strong civil society backing was also vital to exerting political pressure on governments to adopt inclusive and participatory approaches in global health initiatives, such as the Global Fund to Fight AIDS, Tuberculosis and Malaria. CSOs played a crucial role in advocating for the inclusion of affected communities in the decision-making processes and the implementation of Global Fund programs (166). For example, in Ukraine, CSOs successfully lobbied for the inclusion of harm reduction strategies and the participation of people who inject drugs in the country's Global Fund grant proposals (167). Moreover, civil society engagement has been essential in promoting accountability and transparency in the Global Fund's operations. CSOs have actively participated in the Global Fund's governance structures, such as the Country Coordinating Mechanisms, and have advocated for the incorporation of community-based monitoring and evaluation systems (168). These efforts have contributed to the Global Fund's commitment to engaging civil society and affected communities as key partners in the fight against HIV/AIDS, tuberculosis, and malaria.

Hence, global health scholars have concluded that despite unequal power dynamics, as non-state actors, CSOs expand critical processes where global decisions are deliberated, supported, and enforced in more democratic, inclusive, and bottom-up ways compared to sole state negotiations (169, 170). However, concerns have been raised about the lack of inclusivity, transparency, and accountability in some high-level decision-making spaces (171–174). Indeed, initial criticism around WHO's first attempts at CSO interactions using web platforms and *ad hoc* invitations during the INB meetings has centered on the narrowness and lack of meaningful participation for those outside formal relations. In a joint open letter to the United Nations High Commissioner for Human Rights, a coalition of 19 organizations and experts expressed deep concern that the Pandemic Treaty process would fail to ensure meaningful civil society participation, particularly for those most impacted by unequal COVID-19 responses and those playing a fundamental role in ensuring an effective pandemic response (175). The Civil Society Alliance for Human Rights in the Pandemic Treaty further highlighted that the INB's proposed modalities of engagement for non-state actors severely and unjustifiably curtailed the ability of civil society organizations, including community-led organizations, to have full, meaningful, and effective participation in the process, as participation would be limited to the small number of organizations in official relations with the WHO (176).

Taken all together, CSOs have historically played a central role in inserting health priorities into political discussions and legal instruments using an array of strategies. Their activities have also spanned phases from agenda-setting to post-negotiation implementation processes nationally. Yet, scholars have acknowledged fewer examples of successful CSO contributions specifically around global agreements with major resource, capacity, and normative implications like binding instruments compared to soft standard-setting frameworks (38). The proposed pandemic accord is the highest-level political commitment to transform pandemic policy, thus remaining a highly complex, uncertain, and contested terrain for civil society navigation. Findings from this paper focusing on perspectives of major groups active around animal welfare aim to elucidate some of these dynamics and tensions.

## 3 Methodology

This research employs a qualitative approach to explore the specific contents related to animal welfare that CSOs recommend for incorporation into the WHO pandemic treaty. The methodology consists of four key phases: data collection, thematic analysis, comparative analysis, and recommendations development.

### 3.1 Data collection

The data collection phase involved a systematic search of the websites and public repositories of three major CSOs actively engaged in the WHO pandemic treaty process: FOUR PAWS, WCS, and A4AH. We used a modified PICO framework to guide our search (that is, Population: Civil Society Organizations; Interest: Animal welfare and health in pandemic prevention; Context: WHO pandemic treaty negotiations). Search terms included combinations of “COVID-19,” “pandemic treaty,” “WHO pandemic agreement,” “animal welfare,” “animal health,” “One Health,” and “zoonotic disease prevention.” The selection criteria focused on documents that specifically addressed animal welfare in the context of the COVID-19 and pandemic treaty, published between 2020 and 2023. We employed purposive sampling (177) to ensure that the selected documents provided substantive inputs on integrating animal welfare and health considerations in the treaty. By doing so, out of 38 initially identified documents, 25 met all inclusion criteria and were selected for analysis. These included formal recommendations to the INB, policy briefs, responses to treaty drafts, and organizational reports. The final corpus collectively spanned 302 pages, representing a comprehensive set of CSO perspectives on the integration of animal welfare and health into the pandemic treaty.

### 3.2 Thematic analysis

In order to capture the full range of CSOs' perspectives without being constrained by preconceived notions, an inductive thematic analysis approach (178) was employed to analyze the collected documents. That is, the analysis allows themes to emerge from the data rather than fitting the data into pre-existing categories. Our analysis was guided by the holistic concept of animal welfare as defined in the Introduction, recognizing its broader scope compared to animal health. This distinction informed our approach to identifying and categorizing CSOs' perspectives on integrating animal welfare into pandemic prevention strategies. Accordingly, the thematic analysis involved systematically coding the collected documents. Two cycles of coding were conducted (179). The first cycle involved open coding, where descriptive labels were assigned to relevant text segments. The second cycle involved axial coding, where the initial codes were refined, consolidated, and grouped into broader categories reflecting recurring themes. The primary coding was conducted by one researcher, an approach that is accepted and supported by the increasing use of thematic analysis in qualitative and/or mixed methods research (180–182), particularly in exploratory studies or when dealing with specialized topics (183). However, to ensure the reliability and validity of the coding process, several measures were implemented. A detailed codebook was developed and regularly updated throughout the coding process, including definitions for each code and examples of text that would fall under each code. The researcher engaged in constant comparison, continuously comparing new data with previously coded data to ensure consistency in code application and to identify potential new themes or subthemes. Besides, peer debriefing sessions were held with co-authors to discuss the emerging codes and themes. These discussions helped challenge assumptions and explore alternative interpretations. Thus, this collaborative approach to analysis enhances the credibility and trustworthiness of the findings. The coding process focused on identifying the key arguments, evidence, and recommendations put forth by the CSOs regarding the integration of animal welfare considerations into various aspects of the pandemic treaty, such as its scope, guiding principles, prevention strategies, governance mechanisms, and policy coherence. Thematic saturation (184) was achieved when no new themes emerged from the data, ensuring that the analysis comprehensively captured the CSOs' perspectives and priorities.

### 3.3 Comparative analysis

Following the thematic analysis, we conducted a comparative analysis. This phase involved selecting the most recent draft of the WHO pandemic treaty available at the time of writing, the “Proposal for the WHO Pandemic Agreement” (128) released in April 2024, for comparison with the themes and recommendations identified from the CSO documents. An article-by-article review of the proposed legal provisions in the April 2024 treaty draft was conducted, assessing the extent to which they aligned with or diverged from the animal welfare considerations and policy priorities advocated by the CSOs. The comparison aimed to identify areas where the treaty drafting process had adequately incorporated the CSOs' perspectives, as well as aspects that were overlooked or required further strengthening. To *supplement* (although at the risk of oversimplifying) the analysis, we developed a custom comparative framework specifically for the study. The framework was inspired by policy assessment approaches that utilize subjective scoring systems, drawing on methods from various fields of policy research (185–187). We designed a subjective scoring system on a scale of 0–10 for both alignment and divergence, allowing for a nuanced representation of the degree of concordance between CSOs' perspectives and treaty provisions. The development of this scale involved several steps. We began with an initial scale design based on the research team's expertise in international law, public health, and animal rights. This was followed by pilot testing of the scale on a sample of treaty articles and CSOs' perspectives. Based on the pilot results and team discussions, we refined the scale further. To ensure reliability, we conducted a validation process through independent scoring by two researchers, followed by comparison and reconciliation of any discrepancies. In the final scoring system, alignment scores were interpreted as follows: scores of 0–2 indicated minimal to no alignment, 3–5 suggested partial alignment, 6–8 represented good alignment, and 9–10 denoted excellent alignment. Similarly, for divergence, scores of 0–2 indicated minimal to no divergence, 3–5 suggested partial divergence, 6–8 represented significant divergence, and 9–10 denoted major divergence. Nevertheless, it is highly important to note that this framework and the resulting scores are inherently subjective, based on our interpretation of the degree of alignment between CSOs' perspectives and treaty text. Therefore, the scores should be merely viewed as indicative rather than definitive measures.

### 3.4 Recommendations development

Based on the insights gained from the gap analysis, a set of targeted recommendations was developed to address the identified shortcomings and align the treaty more closely with CSOs' perspectives. These recommendations aimed to guide policymakers and treaty negotiators toward a more comprehensive and effective integration of animal welfare considerations into the final text of the WHO pandemic treaty and its implementation frameworks.

Yet, let us be clear about one thing concerning researcher positionality. In conducting this research, we acknowledge the influence of our diverse academic and professional backgrounds in international law, public health, and animal rights on our approach and interpretations. Our collective expertise in these fields provides valuable insights but may also predispose us to certain biases, particularly in prioritizing animal welfare and One Health approaches. In response, we have made conscious efforts to consider diverse cultural and geographical perspectives throughout our analysis and engaged in regular team discussions to challenge our assumptions. By maintaining a reflexive approach (188), we aim to provide a balanced analysis while recognizing the inherently interpretive nature of our assessments. We invite readers to critically engage with our findings, considering how our backgrounds may have shaped the research outcomes.

## 4 Findings: thematic analysis of CSO perspectives

The primary objective behind the pandemic treaty is to fill existing gaps in current frameworks and institute a more coordinated global approach toward tackling future disease outbreaks with pandemic potential (5). To ensure that these goals are achieved comprehensively, the treaty must integrate perspectives from diverse actors (147, 148). Key CSOs working at the intersection of animal welfare, conservation, and health have an essential role to play in this process. Their positions on and recommendations for an effective, equitable treaty provide important insights that member states can draw upon. This section focuses specifically on the contributions of three major CSO groups—FOUR PAWS, WCS, and A4AH—that have engaged substantively with the treaty process. It analyzes submissions made by these groups between 2020–2023 and examines the key themes related to animal welfare that emerge from them. Five major thematic areas form the basis of analysis: (1) prevention of zoonotic spillover, (2) One Health approach, (3) animal health systems and infrastructure, (4) sustainable and ethical animal management practices, and (5) policy coherence and governance (see Table 2).

**Table 2:** Key themes on integrating animal welfare into the pandemic treaty.

**Themes**	**Codes**	**Examples**
Prevention of Zoonotic Spillover	Primary prevention; Root cause identification; High-risk interface reduction; Precautionary approach; Integrated surveillance; Addressing anthropogenic drivers; Land use change and deforestation; Commercial wildlife trade; Intensive animal agriculture; Live animal markets; Climate change	- “Measures to prevent spillover of pathogens at the human-animal-environment interface are neither captured in the draft, nor in any existing legal instruments” (FOUR PAWS, *Remarks on the Conceptual Draft*, 2022). - “It is imperative that we address these issues with interventions such as policies that limit forest conversion and stop the commercial trade and consumption of live wild birds and mammals” (WCS, *Pandemic Prevention at Source and a Multilateral Solution*, 2022). - “The substantive content of the accord must include provisions to stop spillover of zoonotic disease from animals to people in the first place” (A4AH, *Recommendations to the INB*, 2022).
One Health Approach	Holistic framework; Interconnections between human, animal, and environmental health; Multisectoral collaboration; Community engagement; OHHLEP definition; Non-anthropocentric approach; Upstream drivers of disease emergence; National One Health Action Plans; One Health Joint Plan of Action (OHJPA)	- “Human health institutions will require collaboration with other sectors as has been acknowledged in the Conceptual Zero Draft, therefore Article 17 must be expanded accordingly” (FOUR PAWS, *Remarks on the Conceptual Draft*, 2022). - “While the WHO is the only intergovernmental organization designed and mandated to act across sectors, with human health objectives as a top priority, it is imperative that the WHO actively engage with the other members of the Quadripartite partnership for One Health as well as existing multilateral environment agreements (e.g., CBD, CITES, UNFCCC, CMS) to create synergies on actions to reduce risk of spillover and nature-based solutions” (WCS, *Recommendations on Substantive Elements*, 2022). - “One Health is not a one-way process toward protecting human health only, but takes a holistic approach to optimize the health of humans, as well as animals and ecosystems” (A4AH, *Recommendations to the INB*, 2022).
Animal Health Systems and Infrastructure	Surveillance and disease reporting; Veterinary workforce capacity; Laboratory capacity; Access to veterinary services; Community-based approaches; Early detection; Antimicrobial resistance; Genetic sequencing; Data science; Biosecurity measures; Veterinary medicines and vaccines	- “*Understanding* that animals in poor environments, on poor diets, or under stress increase risks of disease emergence, mutation and transmission, posing threats to global human health” (FOUR PAWS, *Remarks on the Zero Draft*, 2023). - “The Parties will identify and integrate into relevant their pandemic prevention and preparedness plans associated interventions that address the drivers of the emergence and re-emergence of pathogens and disease at the human-animal-environment interfaces, including but not limited to… weak animal health systems and management” (WCS, *Input on the Bureau's text*, 2023). - “The substantive content of the treaty must address prevention of the transmission of zoonotic diseases from animals to people by strengthening animal health systems. Specifically, action must be taken to [i]mprove surveillance systems and capacity to secure early detection of animal disease in wildlife and domestic animal populations” (A4AH, *Recommendations to members states*, 2022).
Sustainable and Ethical Animal Management Practices	Transition from intensive farming; Improving animal welfare; Biosecurity measures; Sustainable food systems; Banning high-risk practices; Natural genetic diversity; Reducing antibiotic use; Animal husbandry regulations; Access to natural environments; Training and capacity building; Paradigm shift in human-animal relationships	- “[T]he risk of pandemics would decrease if improving animal welfare was a central aspect of pandemic prevention plans” (FOUR PAWS, *Report on Preventing Pandemics*, 2022). - “Poor animal welfare in food systems facilitates transmission of disease and AMR. This includes poor animal care, poor biosecurity, unsustainable wildmeat harvesting, and agricultural encroachment on wildlife. Animal-based food systems are a bigger driver of zoonosis events than the wildlife trade” (A4AH, *Advisory note to inform Pandemic Instrument negotiations*, 2023).
Policy Coherence and Governance	Intersectoral collaboration; Coherence between international agreements; National coordination mechanisms; Role of Quadripartite; Sustainable financing; Whole-of-government approach; Whole-of-society approach; International institutions support; Data sharing and interoperability; Cross-sectoral policies	- “Protecting public health and achieving health for all are the ultimate outcomes, which will require collaboration among stakeholders involved in protecting human, animal and environmental health” (FOUR PAWS, *Brief on Article 5*, 2023). - “A national focal point would coordinate across all relevant ministries on efforts (e.g., implementation, mitigation, reporting) in a trans-sectoral, One Health approach to human, animal, and environmental health” (WCS, *Recommendations on Substantive Elements*, 2022).

### 4.1 Prevention of zoonotic spillover

The first major theme that emerges from the documents of the three CSOs is the critical importance of preventing zoonotic spillover from animals to humans as the primary strategy for avoiding future pandemics. All three groups emphasize that pandemics arise when pathogens jump from animals to people, so reducing the risk factors that enable this spillover is paramount. This theme encompasses concepts like root cause identification, high-risk interface reduction, precautionary approach, primary prevention strategies, and integrated surveillance.

FOUR PAWS states in its remarks on the Conceptual Zero Draft that “[t]o fulfill the objectives of the instrument it is necessary to include clear and specific measures to prevent future pandemics including stronger action on the underlying causes of pandemics” and, however, that “[m]easures to prevent spillover of pathogens at the human-animal-environment interface are neither captured in the draft, nor in any existing legal instruments” (189). Thus, the organization argues that provisions for prevention, particularly primary prevention, must be at the core of the treaty to fill current policy gaps (189). Similarly, WCS issued a statement affirming that “pandemics of zoonotic origin, such as COVID-19, are directly related to the increased human/wildlife interface caused by land-use change, particularly destruction of intact ecosystems” and that “it is imperative that we address these issues with interventions such as policies that limit forest conversion and stop the commercial trade and consumption of live wild birds and mammals” (190). The group believes that preventing spillover through banning wildlife trade and commercial markets and protecting intact ecosystems is the most effective way to avoid future pandemics (190). A4AH also submitted recommendations stating that since “[t]he root cause of pandemics lies in how pathogens move from animals to people,” “the substantive content of the accord must include provisions to stop spillover of zoonotic disease from animals to people in the first place” through measures like “[i]mprov[ing] surveillance systems and capacity...at critical points like farms, border crossings and wet markets,” and “[i]ncreas[ing] and upskill[ing] the animal health workforce” (191).

The documents point to numerous risk factors that drive spillover, including land use change and deforestation, commercial wildlife trade, intensive animal agriculture, weak animal health systems, live animal markets, and more. For instance, FOUR PAWS notes that reducing behaviors interfering with wildlife, decreasing consumption of animal products, transitioning away from intensive farming, strict regulation of wildlife trade, and ending high-risk practices like fur farming and live animal markets will curb pandemic risk (192). WCS similarly calls for prohibiting commercial wildlife trade and fragmentation of intact forests (190), while A4AH names poor livestock care, unsustainable farming, and wildlife market practices as spillover risks (193).

All three groups advocate for governments to adopt a precautionary approach when formulating policies and obligations aimed at reducing these high-risk human activities and behaviors. For example, FOUR PAWS states that “governments must advocate the following...adopting a highly precautionary approach to risk” regarding wildlife trade (194). WCS likewise references the precautionary principle to justify prohibiting commercial wildlife trade and forest loss even without complete scientific evidence of their risks (195). The CSOs' recommendations for banning commercial wildlife trade and closing live animal markets are particularly relevant in the context of illegal wildlife trade, which often involves poor biosecurity measures, unsanitary conditions, and the mixing of multiple species from different geographical locations, increasing the risk of zoonotic disease transmission. Thus, implementing strong provisions to curb land use change, ban wildlife commercialization, reform high-risk farming methods, improve animal welfare, and strengthen veterinary capacities will significantly reduce opportunities for spillover according to the documents.

### 4.2 One health approach

A second major theme running throughout the documents is the necessity of incorporating a genuine One Health approach that recognizes the complex interconnections between human health, animal health, and environmental health. The groups criticize interpretations of One Health that focus narrowly on human interests at the expense of animal welfare, biodiversity, and ecological integrity. Instead, they promote adopting a holistic One Health framework that aims to foster the wellbeing of humans, animals, and nature in an integrated way.

For instance, FOUR PAWS welcomed the initial inclusion of One Health in the Zero draft of the pandemic accord but stated that “it must be further expanded upon with concrete measures that enable an effective instrument” since “human health institutions will require collaboration with other sectors as has been acknowledged” (189). Thus, the organization argues that successfully preventing pandemics requires moving beyond an anthropocentric, human-centered policy orientation to a genuinely holistic One Health model (196). Similarly, WCS constantly put forth explicate statements endorsing the One Health High-Level Expert Panel's (OHHLEP) definition of One Health (195, 197–200) and calling for the new instrument to make an “[a]ctive engagement with other intergovernmental organizations and existing conventions” like the Convention on Biological Diversity and Convention on International Trade in Endangered Species of Wild Fauna and Flora to “create synergies on actions to reduce risk of spillover and nature-based solutions” using a One Health approach (195). This demonstrates the group's view of One Health as an integrated framework linking biodiversity, conservation, and sustainable development goals alongside pandemic policy. Moreover, A4AH advised that “principles to prevent pandemics should align with OHHLEP's definition of One Health” which “recognizes that One Health is not a one-way process toward protecting human health only, but takes a holistic approach to optimize the health of humans, as well as animals and ecosystems” (191). The coalition affirmed support for the initial ambition to include One Health and prevention per the original WHA decision but cautioned that realizing this ambition requires incorporating animal health systems as part of holistic One Health action (201).

These organizations stress that a truly effective One Health approach must involve multisectoral collaboration, integrate knowledge from various disciplines, engage communities at the human-animal-environment interface, and address upstream drivers of disease emergence. In particular, WCS advocates for the need to “break down sectoral and disciplinary silos and foster much needed cooperation and collaboration” to effectively implement a One Health approach. Also, these organizations emphasize that One Health is not merely a concept but a call to action, the priority of which is to develop and implement National One Health Action plans to operationalize One Health principles and guide pandemic prevention efforts (192). In this regard, they refer to the One Health Joint Plan of Action (OHJPA) as a practical framework for translating the One Health concept into concrete actions. For instance, FOUR PAWS states that “[t]he One Health Joint Plan of Action provides guidance for the operationalization of One Health at national, regional and global levels, and acts as a framework for the development of Member State national action plans” (202). It further recommends that the OHJPA “should be referenced in the negotiating text to serve as a guide on the measures that can be implemented to prevent disease emergence and tackle AMR” (202).

### 4.3 Animal health systems and infrastructure

The documents from FOUR PAWS, WCS, and A4AH consistently emphasize the crucial role of robust animal health systems in pandemic prevention. They argue that investments in animal health infrastructure, workforce capacity, and surveillance systems are vital for early detection and control of pathogens in animal populations, ultimately preventing spillover to humans. A4AH underscores this point, stating that “[a]nimal health services are critical to the prevention of disease, the early detection of pathogens, reporting, control, and prevention of spread” (193). However, they note a significant challenge: “[y]et many are under-resourced and cannot comply with the core competencies outlined by the World Organization for Animal Health” (193). This underinvestment in animal health systems, according to the organizations, is a major driver of zoonotic disease outbreaks and antimicrobial resistance (195). In response, they highlight several key aspects of animal health systems that require strengthening. First of all, surveillance and disease reporting emerge as a critical component. WCS emphasizes the need for “collaborative surveillance” that encompasses “the health of the environment, ecosystems, and animals” to enable prevention and responses supporting ecological integrity alongside societal needs (203). A4AH echoes this sentiment, recommending “improving surveillance systems and capacity to secure early detection of animal disease in wildlife and domestic animal populations, and ensure the ability to respond effectively, from the community level to the global level” (191, 201).

Equally important is the capacity of the veterinary workforce and laboratory. A4AH stresses that “[s]killed frontline workers play a vital role in building the resilience of communities and health systems to respond to threats, including the detection, prevention and treatment of zoonotic diseases in wildlife and domestic animals” (201). It, therefore, advocates for efforts to “[i]ncrease and upskill the animal health workforce” to prevent zoonoses through better detection, reporting, and rapid response to outbreaks in animals before they spill over to humans (201). In its line-by-line edits and comments on the WHO CA+ zero draft, FOUR PAWS starts to clearly recommend the paralleling position of the veterinary workforce with the health and care workforce (under the framework of Article 12) (194). In *WCS input on the Proposal for negotiating text of the WHO Pandemic Agreement*, it adds explicitly “including veterinary health workers” to Article 7 (Health and care workforce), treating these workers as one type of “a skilled, trained, competent, and committed health and care workforce” (203). In addition to the workforce, veterinary laboratory capacity is another crucial aspect highlighted in the documents. For instance, while WCS supports, in general, strengthening laboratory capacity to identify and assess the risks and emergence of pathogens and variants with pandemic potential, it specifically emphasizes the need to “strengthen and maintain veterinary and public health laboratory and diagnostic capacities, especially with respect to the capacity to perform genetic sequencing, data science to assess the risks of detected pathogens and to safely handle samples containing pathogens, and the use of related digital tools” (203). Likewise, yet more emphasis on the promotion and implementation of the One Health approach, A4AH suggests strengthening “integrated” surveillance system and “public health and verrinary” laboratory capacity (204).

Access to veterinary services, particularly in remote or resource-limited settings, is also identified as a key component of robust animal health systems. A4AH emphasizes the need to “[p]rovide access to good quality veterinary medicines and vaccines, and ensure animal health professionals have the skills to use them properly, to prevent zoonotic diseases and to reduce the risk of antimicrobial resistance” (191). A4AH also highlights the importance of community-based approaches, recommending to “[i]ncrease participation of community animal health workers and community members, especially rural and marginalized communities (like pastoralists who regularly interact with animals), as sentinels of surveillance for monitoring animal health in their local areas” (191).

### 4.4 Sustainable and ethical animal management practices

A fourth theme woven throughout the documents is the intrinsic linkage between animal welfare and sustainable management practices. FOUR PAWS, WCS, and A4AH collectively argue that upholding animal welfare is crucial both for ethical reasons and for creating resilient, healthy systems that benefit both animals and humans in the long term. For instance, FOUR PAWS observes in its 29 in-depth telephone interviews on the future of human-animal relationship after COVID-19 that respondents connect poor animal welfare conditions, such as high stock numbers and close proximity of animals within farms, to higher risks of diseases spreading among animals and zoonoses as well as increased use of antibiotics in farming which furthers pathogen resistance (192). The group elsewhere recommends that governments “put in place mechanisms to limit the disturbance, unnatural migration, and removal of wild animal species through human encroachment” including unsustainable food systems to curtail spillover risks (205). In parallel, WCS proposes adding language to the Zero draft on optimizing animal health and welfare as part of One Health strategies, minimizing antimicrobial usage through improving livestock care, and enhancing biosecurity in animal production to reduce pathogen transfers (199). The organization previously issued statements warning that animal welfare declines with intensive, industrialized agriculture and that subsequent heavy antibiotic use propels antimicrobial resistance, imperiling animal, and human health (198). Additionally, A4AH cautions that poor animal welfare in food systems facilitates disease spread and antimicrobial resistance, while unsound livestock practices drive zoonoses from wildlife (193). It specifically calls for improving animal care to boost immunity and limit antibiotics, as well as increasing livestock efficiency toward better health and lowered ecosystem pressures (191, 201).

Thus, the organizations collectively advocate for a transition away from intensive animal agriculture toward more sustainable and ethical practices. FOUR PAWS recommends “reducing reliance on animal products, shifting away from intensive farming and improving husbandry, preventing human encroachment on wildlife habitats through agricultural land use and deforestation for the production of animal feed” (196). This multifaceted approach aims to address both the welfare concerns and the ecological impacts of current farming practices. To phase out specific high-risk practices that compromise animal welfare, FOUR PAWS specifically calls for “banning fur farms and live animal markets as well as protecting biodiversity and species” (196). A4AH pays attention to the necessity of sustainable food systems that prioritize animal welfare and suggests improving livestock efficiency toward better health and lowered ecosystem pressures, indicating that more sustainable farming methods can simultaneously improve animal welfare and lessen environmental impacts (193). This includes promoting natural genetic diversity in livestock populations rather than relying on high-performance breeding lines that may compromise animal health and resilience (196).

Among the recommendations for sustainable and ethical animal management practices, the organizations emphasize the importance of establishing strong and binding health and welfare regulations and enforcement regarding animal husbandry, transport, market, and slaughter (205). They further advocate for providing animals with access to natural environments and opportunities to express natural behaviors, which can contribute to improved immunity and overall health (205). Besides, training and capacity building for those working with animals is another key recommendation. The organizations call for programs that equip farmers, transporters, and slaughterhouse workers with the knowledge and skills to handle animals humanely and implement proper hygiene and biosecurity measures without compromising animal welfare (196). Ultimately, they actually envision a paradigm shift in how humans view and interact with animals by promoting empathy, understanding, and respect for animals in all contexts, recognizing their intrinsic value beyond their utility to humans. This approach aligns with the holistic principles of the One Health approach, recognizing the interconnectedness of human health, animal welfare, and environmental integrity in addressing global health challenges.

### 4.5 Policy coherence and governance

The fifth theme evident across the documents is the necessity of intersectoral policy coherence and collaborative governance embracing veterinary, environmental, development, and health authorities in an integrated One Health approach to avoid pandemics through prevention at source. The groups highlight the lack of coordination across government and knowledge sectors as hampering progress on spillover prevention and global health security via fragmented policies, financing, and implementation. They argue that deep collaboration and cooperation are essential for the effective implementation of sustainable animal management practices and One Health strategies. For instance, FOUR PAWS underlines that animal and environmental health authorities must be involved alongside human health institutions within a pandemic accord since “protecting public health and achieving health for all are the ultimate outcomes,” which “will require collaboration among stakeholders involved in protecting human, animal and environmental health” (206). The organization argues that “truly prevent future global pandemics” obliges “stronger collaboration among human, animal and environmental health partners” in designing and implementing health policies (207).

A recurring point made by these CSOs' documents is the importance of ensuring coherence between the new pandemic instrument and existing international agreements relevant to pandemic prevention. FOUR PAWS emphasizes the need for “legal coherence between existing environmental and animal-related treaties such as the CBD, CMS and CITES” and asserts that the new instrument “should reinforce and complement their existing provisions in line with a One Health approach” (205). Alignment with the “UN Convention against Transnational Organized Crime (UNTOC)” (205) and coherence with the sanitary and phytosanitary (SPS) annex of the WTO agreement (194) are also what FOUR PAWS urges governments to take into account in the treaty drafting process. For the latter WTO agreement, FOUR PAWS particularly notes that such coherence “allows member states to take the necessary measures for the protection of human, animal, or plant health, as long as the measures taken are not inconsistent with the WTO” (194). Likewise, WCS calls for “[a]ctive engagement with other intergovernmental organizations and existing conventions” to create synergies on actions to reduce risk of spillover and nature-based solutions (195). WCS specifically mentions aligning the pandemic accord with “existing multilateral environment agreements (e.g., CBD, CITES, UNFCCC, and CMS)” (195).

At the national level, the organizations recognize the critical role of strong national coordination mechanisms. WCS advocates for designating national focal points for pandemic prevention, stating that “such a national focal point would coordinate across all relevant ministries on efforts (e.g., implementation, mitigation, reporting) in a trans-sectoral, One Health approach to human, animal, and environmental health. Each State Party would be required to designate or establish a national focal point for pandemic prevention-related communications with WHO and relevant sectors within the country, as well as other Member States and intergovernmental organizations, where appropriate” (195). FOUR PAWS supports “a whole-of-government coordination mechanism involving relevant departments, ministries and institutions, and a whole-of-society approach involving relevant stakeholders, expert institutions and communities at the human-animal-environment interface” (208). FOUR PAWS believes that this approach is essential “to enable them to transition away from high risk practices and protect themselves” (208).

Another key point shown in the CSOs' documents refers to leveraging the expertise and mandate of international institutions, particularly the Quadripartite, to provide essential support for national One Health efforts. FOUR PAWS argues that “[t]he active role of all four Quadripartite Institutions in preparing internationally implementable guidelines and standards and supporting Member States in meeting their One Health commitments must be reflected” in the pandemic instrument “to ensure Member States have access to expert and technical support as needed to successfully develop and implement robust One Health strategies” (202). WCS clarifies that “[t]he role of the Quadripartite must be explicitly reflected in the proposed governance of the WHO CA+, especially given the recent release of their One Health Joint Plan of Action and its relevance for pandemic prevention” (197). It also urges that while “[t]he WHO should retain its central role, but it must work closely with the Food and Agriculture Organization of the United Nations (FAO), the World Organization for Animal Health (WOAH) and the United Nations Environment Programme (UNEP)” (197). This collaboration is seen as “crucial to ensure that the WHO CA+ moves beyond the skewed, anthropocentric approach to health which has failed us in the past and reflects the multisectoral, comprehensive approach needed to protect us from future pandemics” (197).

To summarize, five major themes emerge from the CSOs' documents regarding integrating animal welfare within a new pandemic accord. These themes demonstrate broad agreement among the CSOs that current human interactions with wild and domestic animals, including land use change, wildlife trade, intensive animal agriculture, and live animal markets, significantly increase the risk of pandemic emergence and spread. The organizations advocate for transformative changes in these human-animal relationships. Their recommendations include banning commercial wildlife trade, transitioning away from intensive farming practices, improving animal welfare standards, strengthening biosecurity measures, reducing antimicrobial use, and enhancing community-based animal health capacities. Significantly, the documents strongly emphasize that fragmented or reactive approaches focusing solely on post-spillover responses or narrow preparedness measures will be insufficient to address the complex challenges of pandemic prevention. The COVID-19 pandemic has starkly illustrated the need for a paradigm shift from reactive “symptom control” to proactive prevention strategies that address the root causes of zoonotic spillover. This shift requires a fundamental transformation in how human societies interact with animals and the environment, guided by a holistic One Health framework that prioritizes the wellbeing of humans, animals, and ecosystems alike. Thus, the CSOs provide comprehensive recommendations, evidence, and models for this transformative approach. They call for coordinated action across sectors, shared governance mechanisms, sustainable financing, and alignment of international policies to create an integrated framework for addressing global health challenges. These proposals await serious consideration and implementation by governments, development agencies, communities, and other stakeholders.

## 5 Discussion: comparative analysis of CSO perspectives and treaty drafting

**Figure 1:**
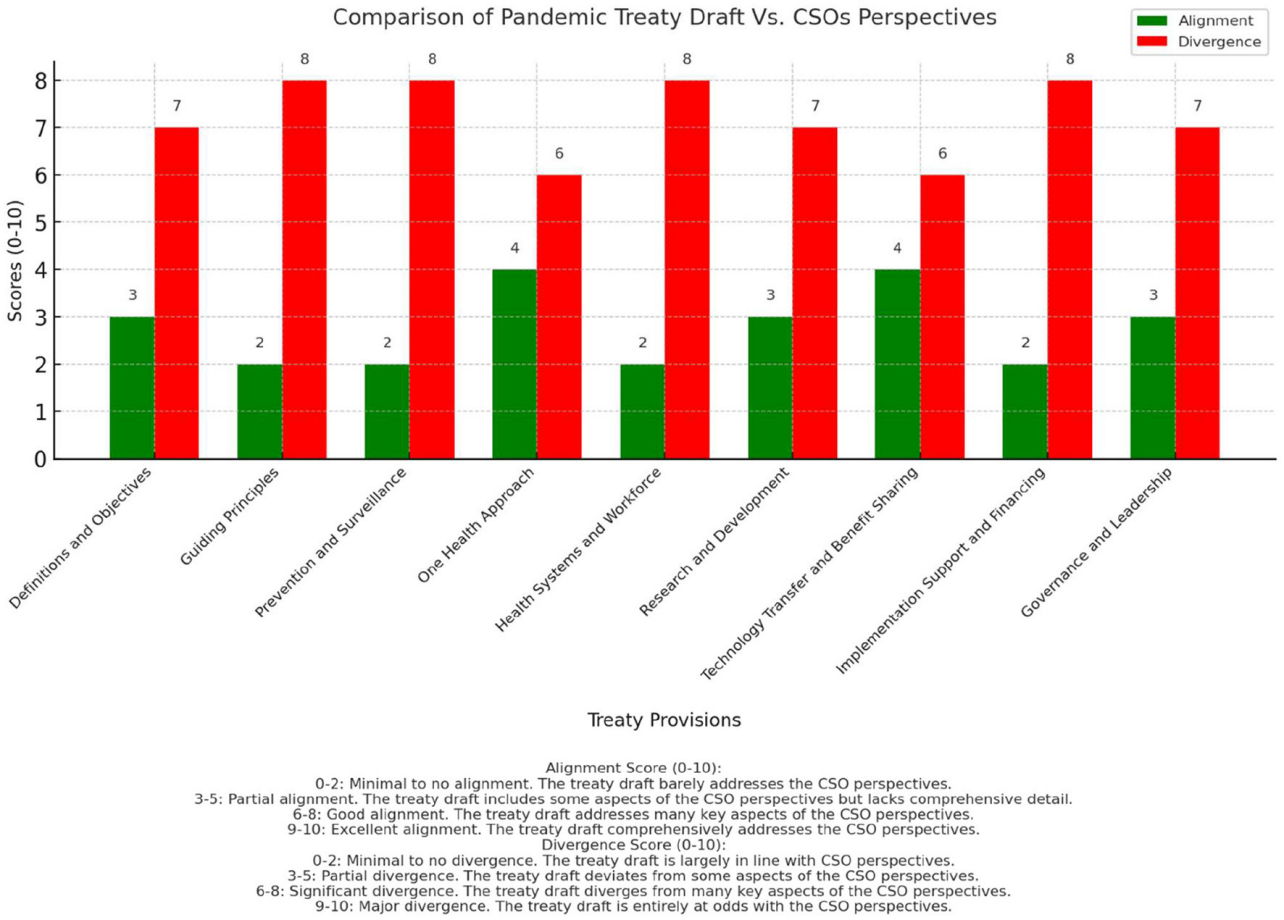
Comparison of pandemic treaty draft vs. CSOs perspectives.

In this section, we examine the “Proposal for the WHO Pandemic Agreement” (128), dated 22 April 2024, and provide a comparative analysis with the key perspectives and recommendations put forth by CSOs like FOUR PAWS, WCS, and A4AH. The comparison focuses on the five key themes identified in section 4: (1) prevention of zoonotic spillover, (2) One Health approach, (3) animal health systems and infrastructure, (4) sustainable and ethical animal management practices, and (5) policy coherence and governance. Using the subjective scoring system described in our methodology (Section 3.3), we assess the degree of alignment and divergence between CSO recommendations and the treaty draft for each theme. Table 3 provides a detailed breakdown of this analysis, including specific examples from the treaty draft and identified gaps. Figure 1 offers a visual representation of the subjective scores across all themes. Our analysis reveals varying degrees of alignment across different aspects of the treaty draft, with some areas showing good incorporation of CSOs' perspectives and others demonstrating significant gaps.

**Table 3:** Details of the comparative analysis between CSOs' perspectives and treaty draft status.

**Treaty Provisions**	**CSOs' Perspectives**	**Treaty Draft Status**	**Comparison Analysis**	**Identified Gaps**	**Themes Related**
Definitions and Objectives (Articles 1–2)	Define key One Health concepts including animal welfare, wildlife conservation, and sustainable food systems. Broaden scope to encompass transformative, integrated approaches targeting root drivers.	Article 1(b) defines One Health as “an integrated, unifying approach that aims to sustainably balance and optimize the health of people, animals and ecosystems.” Article 2 states the treaty objective is “to prevent, prepare for and respond to pandemics.”	Progress in One Health definition, but lacks specific definitions for animal welfare, wildlife conservation, and sustainable food systems. Objective focuses on reactive measures over proactive reforms to human systems and behaviors that put animal welfare at risk.	Lack of comprehensive definitions for key animal welfare and conservation concepts. Absence of sustainable governance in the treaty's objectives.	Prevention of zoonotic spillover; One Health approach; Sustainable and ethical animal management practices; Policy coherence
Guiding Principles (Article 3)	Articulate animal welfare, conservation, and the precautionary approach as guiding principles.	Article 3 outlines guiding principles including sovereignty, human rights, equity, solidarity, and science-based decision making.	Principles lack explicit articulation of animal welfare or rights, biodiversity safeguarding, or adopting precautionary policies. Focus primarily on human-centric approaches.	No parallel in principles for animal welfare or rights. Absence of precautionary principle and conservation imperatives.	Prevention of zoonotic spillover; Sustainable and ethical animal management practices; Policy coherence
Prevention and Surveillance (Article 4)	Legally bind risk reduction interventions on wildlife trade/markets and agricultural practices. Implement science-based measures to curb threats from risky human practices.	Article 4 (Pandemic prevention and public health surveillance): Each Party shall develop national pandemic prevention and public health surveillance plans that cover zoonotic spill over and spillback prevention.	The treaty mentions zoonotic spillover prevention but lacks concrete commitments or specific measures to address wildlife trade, markets, or agricultural practices. Defaults to incremental changes around early detection over substantive obligations confronting root causes.	Absence of binding commitments for specific risk reduction interventions in high-risk areas like wildlife trade and intensive farming. Lack of measures addressing illegal wildlife trade.	Prevention of zoonotic spillover; Sustainable and ethical animal management practices; Animal health systems and infrastructure
One Health Approach (Article 5)	Establish concrete treaty provisions for One Health governance bodies, funding streams, implementation frameworks, and accountability channels.	Article 5 promotes a One Health approach “for pandemic prevention, preparedness and response, recognizing the interconnection between people, animals and the environment.” Commits to identifying and addressing drivers of pandemics at the human-animal-environment interface.	Represents a positive shift toward integrative strategies, but lacks specific obligations or mechanisms institutionalizing genuine cooperation. Risks enabling further siloed, *ad hoc* approaches. Lacks binding commitments within the core text for structural and policy integration.	Lack of specific mechanisms for cross-sectoral cooperation and integrated One Health implementation. Absence of consolidated governance bodies and funding streams for One Health.	One Health approach; Policy coherence; Prevention of zoonotic spillover
Health Systems and Workforce (Articles 6 and 7)	Formally recognize animal health professionals as vital members of the One Health workforce. Invest in training, infrastructure, equipment, and technologies for animal health systems.	Articles 6 and 7 focus on human infrastructure, personnel, and service delivery components. Commitments to develop resilient health systems and establish a multidisciplinary workforce.	Concentrates almost entirely on human health systems with no equivalent acknowledgments or capacity-building pledges for animal health systems or frontline veterinary workers. Neglects animal disease surveillance, veterinary services, and wildlife monitoring capacities.	Lack of specific provisions for strengthening animal health systems and recognizing animal health professionals. Absence of commitments for veterinary capacity building and animal health infrastructure.	Animal health systems and infrastructure; One Health approach; Prevention of zoonotic spillover
Research and Development (Article 9)	Focus on animal vaccine advancement alongside human prophylactics or therapeutics. Close gaps in animal immunization options.	Article 9 emphasizes scientific collaboration and “open access” approaches for novel findings. Encourages sustained investment, capacity building, and joint ventures.	Lacks equivalent binding commitments to close gaps in animal immunization options. R&D provisions direct benefits overwhelmingly to human populations. Missing commitments for animal vaccine development and research.	Absence of specific provisions for animal health research and vaccine development. Lack of dedicated research funding for animal pathogens and zoonoses.	Animal health systems and infrastructure; Prevention of zoonotic spillover; One Health approach
Technology Transfer and Benefit Sharing (Articles 10–12)	Guarantee equivalent dissemination of animal health technologies, vaccines, equipment, and data as part of benefit-sharing obligations.	Articles 10–12 contain language on equity, fairness, and reciprocity in technology transfer and benefit sharing. Establishes a Pathogen Access and Benefit-Sharing (PABS) System.	Omits references to guaranteeing equivalent dissemination of animal health technologies. Focuses near-singularly on human-related diagnostics, therapeutics, information, and capacity diffusion. PABS system does not delineate governance for animal pathogens.	Lack of specific provisions for animal health technology transfer and benefit sharing. Absence of mechanisms to ensure animal health products access in lower-resourced settings.	Animal health systems and infrastructure; Policy coherence; One Health approach
Implementation Support and Financing (Articles 19 and 20)	Allocate specific funding for animal health, wildlife conservation, and environmental projects. Mandate equitable resource allotments across human, animal, and environmental preparedness.	Articles 19 and 20 offer broad pledges to assist capacity building but lack targeted commitments for animal and environmental health initiatives. Establishes a Coordinating Financial Mechanism.	No provisions allocating specific minimum percentages, quantities, or conditionalities for pooled funds to flow to animal health, wildlife conservation, or environmental projects. Focuses primarily on supporting human health institutions and initiatives.	Absence of dedicated funding streams for animal and environmental health initiatives. Lack of specific financial commitments for One Health implementation.	Animal health systems and infrastructure; Policy coherence; One Health approach
Governance and Leadership (Articles 21–25)	Establish permanent One Health governance councils with equitable representation from WHO, WOAH, UNEP, and FAO.	Articles 21–25 center WHO's authority without corresponding empowerment of WOAH, UNEP, or FAO in collaborative decision-making and coordination. Establishes a Conference of Parties and WHO Secretariat as primary bodies.	Lacks parallel leadership or agenda-setting prerogatives for animal and environmental institutions. No rotating arrangements, joint entities, or formal co-leadership roles for FAO, UNEP, or WOAH. Risks entrenching human health-dominated paradigms.	Absence of formal joint governance structures involving animal and environmental health agencies. Lack of integrated dispute settlement mechanisms for One Health issues.	One Health approach; Policy coherence; Animal health systems and infrastructure

### 5.1 Definitions and objectives (Articles 1–2)

The current treaty draft's definitions (Article 1) and objective (Article 2) showcase some alignment with civil society perspectives, particularly regarding the One Health approach (theme 2). The inclusion of a One Health definition in Article 1(b) stating that it “means an integrated, unifying approach that aims to sustainably balance and optimize the health of people, animals, and ecosystems” and “recognizes that the health of humans, domestic, and wild animals, plants and the wider environment (including ecosystems) is closely linked and interdependent” represents progress toward the holistic framing consistently advocated by groups like FOUR PAWS, WCS, and A4AH. This definition signals growing acknowledgment of the complex interconnections between human, animal, and environmental health that CSOs argue must inform pandemic policy.

However, the treaty draft still lacks specific definitions of other key One Health concepts emphasized by civil society, such as sustainable and ethical animal management practices (theme 4), wildlife conservation, and sustainable food systems, which are crucial for preventing pathogen spillover (theme 1). The absence of clear, internationally agreed-upon understandings of these terms within the operative text risks ambiguity during national interpretation and implementation phases. CSOs contend that explicitly articulating definitions would provide important guidance for governments on upholding animal health as part of pandemic prevention obligations.

Moreover, while Article 2 states that the treaty objective is “to prevent, prepare for and respond to pandemics,” this fails to sufficiently broaden the scope to encompass transformative, integrated approaches targeting root drivers like unsustainable animal-human interactions, which is essential for policy coherence (theme 5). The phrasing remains focused primarily on reactive measures over proactive reforms to human systems and behaviors that put animal welfare at risk and propel pathogenic emergence, such as habitat degradation, wildlife exploitation, and intensive farming. CSOs assert that effectively fulfilling the prevention goal requires directly acknowledging animal and environmental domains as inextricable to avoiding future outbreaks.

Based on our analysis, we assign the following scores for this section:alignment score: 3/10 divergence score: 7/10. These scores reflect the partial inclusion of One Health concepts but significant gaps in addressing specific animal welfare and sustainable management practices. The low alignment and high divergence scores indicate that while there is some acknowledgment of interconnected health systems, the treaty's definitions and objectives fall short of the comprehensive approach advocated by CSOs.

### 5.2 Guiding principles (Article 3)

The guiding principles enshrined under Article 3 include notable human rights language and scientific evidence promotion but largely omit equivalent sustainable and ethical animal management practices (theme 4) and conservation imperatives or precautionary approaches that CSOs highlight as vital for steering treaty implementation and preventing pathogen spillover (theme 1). Article 3(2)'s commitment to upholding “full respect for the dignity, human rights and fundamental freedoms of all persons, and the enjoyment of the highest attainable standard of health of every human being” and Article 3(6)'s affirmation of “the best available science and evidence as the basis for public health decisions” resonate to some extent with civil society calls for rights-based, evidence-informed foundations.

Yet, no parallel exists in the principles explicitly articulating animal welfare or rights, biodiversity safeguarding, or adopting precautionary policies even absent complete data. Leading groups like FOUR PAWS have repeatedly urged negotiators to formally recognize animal wellbeing as a core ethical obligation and practical necessity for pandemic prevention, citing linkages between poor livestock conditions, wildlife mistreatment, and zoonotic pathogen spillover. WCS and others contend the principles must also unambiguously embrace conservation and ecological integrity as fundamental to lowering anthropogenic threats. The omission of the precautionary principle, which justifies early interventions to mitigate plausible risks without requiring definitive proof, overlooks CSO arguments that this approach has precedent in environmental treaties and represents a crucial paradigm shift beyond reactive conventions.

Thus, we assign the following scores for this section: alignment score: 2/10; divergence score: 8/10. These scores demonstrate the significant gap between the CSOs' recommendations and the treaty's guiding principles. The low alignment score indicates that the treaty fails to incorporate key animal welfare and conservation principles advocated by CSOs. The high divergence score highlights the treaty's focus on human-centric approaches, overlooking the interconnected nature of human, animal, and environmental health in pandemic prevention and response.

### 5.3 Prevention and surveillance (Article 4)

The treaty's Article 4 centers overwhelmingly on developing public health surveillance, laboratory biosafety, and infection control capacities. Although Article 4(2)(f) references “zoonotic spill over and spillback prevention” as part of national pandemic prevention plans, it lacks any concrete direction or binding commitments for governments to implement science-based measures to demonstrably curb clearly identified threats from risky human practices like wildlife trade, live animal markets, biodiversity loss, or intensive livestock production (theme 1). Despite the extensive evidence provided by CSOs on the necessity of meaningful policy reforms across these domains, Article 4 defaults to only incremental changes around early detection over substantive obligations confronting root causes, undermining efforts to strengthen animal health systems and infrastructure (theme 3) and policy coherence (theme 5). Moreover, the current treaty draft lacks specific provisions addressing illegal wildlife trade and its associated risks. Given the significant role of illegal wildlife trade in facilitating pathogen spillover, the absence of targeted measures to combat this practice represents a major gap in the treaty's prevention and surveillance efforts.

Leading conservation groups like WCS have consistently called for the treaty to legally mandate restrictions or bans on commercial trade in wild birds and mammals, close unhygienic markets selling live wildlife, and protect intact ecosystems from human encroachment linked to viral emergence. Animal welfare organizations like FOUR PAWS have similarly advocated for binding commitments to end factory farming methods such as extreme crowding and widespread antibiotic usage that facilitate pathogen evolution and transmission. Upholding the precautionary principle, CSOs argue even absent definitive proof, the high plausibility and catastrophic consequences of pandemics spreading through these channels justifies codifying policy changes that redesign human interactions with domestic and wild animal populations to prioritize collective health.

In light of our evaluation of this provision against CSOs' perspectives, which reveals a substantial disparity, the alignment scores a mere 2 out of 10. Conversely, the divergence is stark, rating 8 out of 10. These scores underscore the treaty draft's failure to adequately address the root causes of zoonotic spillover and its overemphasis on reactive measures rather than proactive prevention strategies advocated by CSOs.

### 5.4 One health approach (Article 5)

Article 5 of the treaty draft contains language promoting a One Health approach (theme 2) “for pandemic prevention, preparedness and response, recognizing the interconnection between people, animals and the environment” through “coherent, integrated, coordinated and collaborative” actions “among all relevant organizations, sectors and actors.” The Parties also commit in Article 5(2) “to identify and address the drivers of pandemics and the emergence and re-emergence of disease at the human-animal-environment interface through the introduction and integration of interventions into relevant...plans,” as well as to implement national One Health policies, community engagement, and joint workforce trainings under Article 5(3).

While this represents a positive shift in the direction of integrative, systems-based strategies that CSOs champion, the Article still lacks specific obligations or mechanisms institutionalizing genuine cooperation. Groups like WCS and A4AH have highlighted the need for concrete treaty provisions establishing consolidated One Health governance bodies, funding streams, implementation frameworks, and accountability channels to drive transformative coordination across currently fragmented animal, human, and environmental authorities. Although Article 5(4) references “modalities, terms and conditions and operational dimensions” of One Health “shall be further defined in an instrument” to become operational by 31 May 2026, CSOs assert binding commitments within the core text itself are vital for propelling long overdue structural and policy integration matching the multidimensional nature of pandemic threats. Otherwise, the Article risks enabling further siloed, *ad hoc* approaches under aspirational headers misaligned with scientific realities and on-the-ground needs, which fails to ensure policy coherence (theme 5).

The One Health approach in Article 5 shows promise but falls short of CSO expectations. While it acknowledges the interconnectedness of human, animal, and environmental health, it lacks concrete mechanisms for implementation. Therefore, this partial alignment with CSO recommendations earns a score of 4 out of 10. The divergence, stemming from the absence of specific, binding commitments and governance structures, rates a 6 out of 10.

### 5.5 Health systems and workforce (Articles 6–7)

Articles 6 and 7 on preparing resilient health systems and multidisciplinary workforces for pandemic prevention and response concentrate almost entirely on human infrastructure, personnel, and service delivery components. The provisions extensively detail commitments around bolstering “scalable clinical care, quality routine and essential health care services,” “laboratory and diagnostic capacities,” “social and behavioral sciences,” and “human resources for health” to enable robust responses. Yet, no equivalent acknowledgments or capacity-building pledges appear in these critical Articles for chronically neglected animal health systems and infrastructure (theme 3) or frontline veterinary workers that civil society emphasizes as equally indispensable for early zoonoses detection and containment (theme 1).

The Articles' prevailing focus on preparing for post-spillover scenarios in human populations with scarce reference to strengthening animal disease surveillance, veterinary services, livestock biosecurity, or wildlife monitoring capacities that could prevent those spillovers from occurring at all exemplifies ongoing asymmetries. CSOs like A4AH have repeatedly urged treaty negotiators to redress these gaps by formally recognizing animal health professionals—from veterinarians to community animal health workers—as vital members of the One Health workforce (theme 2) requiring parallel investment in training, infrastructure, equipment, and technologies. They assert prioritizing resources solely for human systems while continuing to neglect animal and environmental dimensions will perpetuate hazardous weaknesses enabling undetected pathogen transmission and amplification. Proposals to expand provisions to incorporate language committing support for animal health capacities on equal footing as human competencies remain largely unheeded in the current text, highlighting the lack of policy coherence (theme 5).

Given the above, we find a significant imbalance between human and animal health considerations. The strong focus on human health systems, while important, overshadows the crucial role of animal health in pandemic prevention. This skewed emphasis results in a low alignment score of 2 out of 10 with CSO recommendations. The divergence from a truly integrated One Health approach is notable, warranting a high score of 8 out of 10.

### 5.6 Research and development (Article 9)

Article 9′s emphasis on promoting scientific collaboration and “open access” approaches for novel findings reflects broader trends in research and development (R&D) debates. It encourages sustained investment, capacity building, joint ventures, clinical trial coordination, and “reduced” licensing costs for publicly financed medical countermeasures. However, CSO recommendations around R&D provisions focusing on animal vaccine advancement alongside human prophylactics or therapeutics are comparatively sparse, despite the crucial role of animal health systems and infrastructure (theme 3) in preventing pathogen spillover (theme 1).

Missing are equivalent binding commitments to close massive gaps in animal immunization options, especially for priority pathogens circulating in livestock and wildlife populations across developing regions. Existing animal vaccine coverage, accessibility mechanisms, and dedicated research funding pale in comparison to human-focused streams, weakening a crucial tool for curbing viral circulation and evolution before human transmission (209). Rectifying this imbalance through earmarked support represents a leading civil society priority to expand the concept of universal health coverage to include animal disease prevention, yet remains largely unaddressed in the draft. If R&D provisions continue to direct benefits overwhelmingly to human populations at the expense of attention to immunological interventions for key animal reservoirs, they risk reinforcing the lack of preparedness at the source and undermining policy coherence (theme 5).

Taking account of the above, the treaty's approach to research and development demonstrates a concerning lack of balance between human and animal health priorities. Although it makes strides in promoting scientific collaboration, it falls short in addressing animal health research needs. Thus, this partial alignment with CSO recommendations results in a score of 3 out of 10. Correspondingly, the divergence from a comprehensive One Health research agenda is significant, scoring 7 out of 10.

### 5.7 Technology transfer and benefit sharing (Articles 10–12)

Articles 10 through 12 contain notable language reflecting CSO inputs around equity, fairness, and reciprocity in the context of transferring technology, know-how, and benefits associated with pandemic-related products. Article 10(1) obliges Parties “to achieving more equitable geographical distribution and scaling up of the global production of pandemic-related health products and increasing sustainable, timely, fair and equitable access to such products, as well as reducing the potential gap between supply and demand during pandemics, through the transfer of relevant technology and know-how on mutually agreed terms.” Article 11 further elaborates on facilitating transfers through “regional or global technology and know-how transfer hubs” and recognizing WTO members' rights to use TRIPS flexibilities for overriding intellectual property in emergencies. Article 12 establishes a novel “multilateral access and benefit-sharing system for pathogens with pandemic potential,” which commits Parties to “rapid, systematic and timely sharing of PABS [the WHO Pathogen Access and Benefit-Sharing System] material and information for...public health risk assessment and, on an equal footing, timely, effective, predictable and equitable access to pandemic-related health products and other benefits, both monetary and non-monetary, arising from such sharing.”

While these provisions align with the spirit of civil society appeals for more equitable distribution of medical resources and knowledge, the Articles omit any reference to guaranteeing equivalent dissemination of animal health technologies, vaccines, equipment, and data as part of benefit-sharing obligations, which is crucial for strengthening animal health systems and infrastructure (theme 3) and preventing pathogen spillover (theme 1). The treaty draft focuses near-singularly on human-related diagnostics, therapeutics, information, and capacity diffusion without clear language on preventing intellectual property or other barriers from likewise hindering animal disease control tools access in lower-resourced settings. The PABS system described also does not delineate specific modalities or governance for materials, information, and innovation associated with animal pathogens. CSOs assert rectifying long-standing asymmetries between human and animal realms remains imperative for manifesting the treaty's equity rhetoric in ways that reach neglected frontiers where threats routinely emerge undetected, yet these disconnects endure, highlighting the lack of policy coherence (theme 5).

The technology transfer and benefit-sharing provisions show a commendable commitment to equity in human health resources but fall short in addressing animal health needs. This partial alignment with CSO recommendations earns a score of 4 out of 10. The divergence, stemming from the neglect of animal health technologies and data in benefit-sharing mechanisms, rates a 6 out of 10. These scores reflect the treaty's progress in human health equity while highlighting the significant gap in animal health considerations.

### 5.8 Implementation support and financing (Articles 19–20)

Articles 19 and 20 on international cooperation for implementation and sustainable financing offer broad pledges to assist capacity building “in all Parties, particularly developing country Parties” but lack targeted commitments or reserved funding streams for animal and environmental health initiatives. While Article 19(1) references “Parties shall cooperate...to sustainably strengthen the pandemic prevention, preparedness and response capacities of all Parties,” the next paragraph clarifies this “shall be facilitated and provided by WHO, in collaboration with relevant organizations” upon request “to fulfill the obligations arising from this Agreement,” which as preceding sections demonstrate, concentrate principally on bolstering human systems.

Similarly, the “Coordinating Financial Mechanism” created under Article 20(3) “is hereby established to provide sustainable financing support, strengthen and expand capacities for pandemic prevention, preparedness and response, and to provide any surge response necessary for day zero, particularly in developing country Parties.” Although its functions include conducting “relevant needs and gaps analyses,” promoting “harmonization, coherence and coordination,” and identifying sources “to support the implementation of this Agreement,” no provisions exist allocating specific minimum percentages, quantities, or conditionalities for these pooled funds to flow to animal health, wildlife conservation, or environmental projects.

CSOs have consistently highlighted that chronically deficient financing for animal and ecological capacities in relation to human investments propels major weaknesses in surveillance, early warning, outbreak containment, laboratory analyses, and frontline service delivery precisely where most disease spillover threats go unnoticed until erupting from local events into international crises. They recommend language explicitly mandating equitable resource allotments across human, animal, and environmental preparedness rather than perpetuating skewed prioritization misaligned with scientific evidence on pandemic origins. The support and financing provisions' prevailing emphasis on country-level assistance for human institutions without equivalent guarantees for animal and ecological funding epitomizes the chasm between One Health rhetoric and operational realities.

Articles 19 and 20 on international cooperation for implementation and sustainable financing offer broad pledges to assist capacity building “in all Parties, particularly developing country Parties” but lack targeted commitments or reserved funding streams for animal health systems and infrastcuture (theme 3), wildlife conservation, or environmental health initiatives, which are essential for preventing pathogen spillover (theme 1) and implementing a genuine One Health approach (theme 2).

While Article 19(1) references “Parties shall cooperate...to sustainably strengthen the pandemic prevention, preparedness and response capacities of all Parties,” the next paragraph clarifies this “shall be facilitated and provided by WHO, in collaboration with relevant organizations” upon request “to fulfill the obligations arising from this Agreement,” which as preceding sections demonstrate, concentrate principally on bolstering human systems.

Similarly, the “Coordinating Financial Mechanism” created under Article 20(3) “is hereby established to provide sustainable financing support, strengthen and expand capacities for pandemic prevention, preparedness and response, and to provide any surge response necessary for day zero, particularly in developing country Parties.” Although its functions include conducting “relevant needs and gaps analyses,” promoting “harmonization, coherence and coordination,” and identifying sources “to support the implementation of this Agreement,” no provisions exist allocating specific minimum percentages, quantities, or conditionalities for these pooled funds to flow to animal health, wildlife conservation, or environmental projects.

The treaty's financing provisions focus primarily on supporting human health institutions and initiatives, without ensuring equivalent investments in animal and ecological domains. This lack of policy coherence (theme 5) in resource allocation undermines efforts to strengthen animal health capacities, which CSOs highlight as crucial for early detection, outbreak containment, and preventing the spillover of zoonotic diseases (theme 1). The support and financing provisions' prevailing emphasis on country-level assistance for human institutions without equivalent guarantees for animal and ecological funding epitomizes the chasm between One Health rhetoric (theme 2) and operational realities. This asymmetry in financing priorities fails to address the root causes of pathogen emergence and amplification at the human-animal-environment interface.

Assessing the implementation support and financing provisions reveals a significant gap between the treaty's approach and CSO recommendations. The lack of specific funding allocations for animal health and environmental initiatives starkly contrasts with CSO calls for equitable resource distribution across all One Health domains. This misalignment results in a low score of 2 out of 10 for alignment with CSO perspectives. The high divergence from a truly integrated One Health financing approach warrants a score of 8 out of 10, underscoring the need for substantial improvements in this area.

### 5.9 Governance and leadership (Articles 21–25)

Lastly, the governance, oversight, and accountability structures envisioned in Articles 21 through 25 heavily center the WHO's ongoing authority without corresponding empowerment of WOAH, UNEP, or FAO in collaborative decision-making and coordination, which is crucial for implementing a genuine One Health approach (theme 2) and ensuring policy coherence (theme 5). Article 21 establishes a Conference of Parties to “regularly take stock of the implementation of the WHO Pandemic Agreement and review its functioning every 5 years” with the ability to “take actions, as appropriate, for the achievement of the objective” and “establish subsidiary bodies.” However, no parallel leadership or agenda-setting prerogatives appear for animal and environmental institutions on core issues like wildlife protection, livestock production, land use planning, climate resilience, and ecological restoration that profoundly influence long-term pandemic risks.

Article 24 further consolidates the WHO Secretariat as the primary body to “perform the functions specified by the WHO Pandemic Agreement, as appropriate, and such other functions as may be determined by the Conference of the Parties or assigned to it under the WHO Pandemic Agreement.” It does not reference any rotating arrangements, joint entities, or formal co-leadership roles for FAO, UNEP, or WOAH to steer critical activities under their technical mandates and comparative advantages. This contrasts with CSO proposals for establishing permanent One Health governance councils that assemble all relevant UN agencies on equitable footing to drive collaborative policies, scientific assessments, strategic planning, and monitoring.

Likewise, the dispute settlement Article 25 specifies Parties “shall seek through diplomatic channels a settlement...including good offices, mediation, or conciliation” rather than designating any shared arbitration forums bridging institutional siloes that frequently undermine coordinated interpretation or enforcement. All these omissions risk entrenching human health-dominated paradigms over purviews like agriculture, trade, environment, land management, and rural development under separate jurisdictional remits, undermining efforts to prevent pathogen spillover (theme 1), strengthen animal health systems and infrastructure (theme 3), and improve sustainable and ethical animal management practices (theme 4).

The governance and leadership provisions of the treaty demonstrate a significant disconnect from the integrated approach advocated by CSOs. The centralization of authority within WHO, without adequate representation from animal and environmental health sectors, reflects a limited understanding of the One Health concept. This approach scores a low 3 out of 10 in terms of alignment with CSO recommendations. The divergence from a truly collaborative, multi-sectoral governance model is substantial, warranting a high score of 7 out of 10.

In summary, comparing the latest treaty draft text with CSO perspectives reveals a decidedly mixed picture (also see Figure 1). While the increased embrace of the One Health language (theme 2) and access and benefit-sharing principles represent notable diplomatic strides, the lack of specific obligations operationalizing transformative policies and investments for animal health systems and infrastructure (theme 3), sustainable and ethical animal management practices (theme 4), and policy coherence (theme 5) exposes significant misalignments between the treaty's soaring rhetoric and still-incrementalist provisions. More notably, sustainable and ethical animal management practices (theme 4) are severely underrepresented throughout the treaty, appearing only tangentially in discussions of prevention and surveillance. This absence highlights a significant oversight in the treaty's approach to comprehensive pandemic prevention. Far from a fundamental rebalancing of human interactions with animals and ecosystems to prioritize the intrinsic linkages between their wellbeing and human health imperatives, the draft agreement perpetuates long-standing reactive conventions around post-spillover preparedness over tackling root anthropogenic drivers (theme 1). The persistent divergence between the text's nominal reformist trappings and its limited obligations around reconstituting the enabling conditions for disease emergence threatens to severely blunt the treaty's impacts.

## 6 Recommendations

The comparative analysis of the WHO pandemic treaty draft and CSO perspectives reveals significant gaps in addressing animal welfare concerns within the global pandemic prevention framework. This section proposes detailed recommendations to bridge these gaps, considering both the practical and ethical implications of integrating animal welfare into a global health instrument.

### 6.1 Addressing key gaps in the treaty draft

To bridge the identified gaps and create a more comprehensive and effective treaty, we propose the following detailed recommendations:

The treaty's definitions and objectives should be strengthened to explicitly recognize the role of animal welfare in pandemic prevention. We recommend expanding Article 1 to include the following definitions: “*(j) ‘Animal welfare' means the physical and mental state of an animal in relation to the conditions in which it lives and dies, as defined by the World Organization for Animal Health*.” and “*(k) ‘Sustainable animal management practices' refers to methods of animal husbandry that meet the physiological and behavioral needs of animals while minimizing environmental impact and zoonotic disease risks*.” These additions align with CSOs' recommendations to include clear definitions of key One Health concepts. Furthermore, Article 2 should be revised to read: “The objective of the WHO Pandemic Agreement, guided by equity and the principles further set forth herein, is to prevent, prepare for and respond to pandemics, *recognizing the critical role of animal welfare and sustainable animal management practices in zoonotic disease prevention*.” This modification addresses the gap identified by, especially, WCS regarding the need for a more holistic approach to pandemic prevention.

To enhance the guiding principles, we propose adding two new principles to Article 3: “*7. the precautionary principle, which allows for protective action before there is complete scientific proof of a risk; this principle shall guide decisions related to practices that may increase zoonotic spillover risks*.” and “*8. respect for animal welfare as an integral component of One Health and pandemic prevention strategies*.” These additions reflect the CSOs' emphasis on the precautionary principle and animal welfare as core ethical obligations.

Article 4 on prevention and surveillance measures should be expanded to include specific provisions addressing wildlife trade and intensive farming practices. We recommend adding: “*4(3) Each Party shall implement measures to regulate and monitor wildlife trade, including: (a) Establishing or strengthening national legislation to combat illegal wildlife trade; (b) Implementing CITES recommendations on reducing zoonotic disease risks in wildlife trade; (c) Enhancing surveillance and hygiene standards in legal wildlife markets*.” Additionally, “*4(4) Each Party shall promote sustainable and ethical animal management practices in animal agriculture, including: (a) Phasing out intensive farming methods that increase zoonotic disease risks; (b) Promoting farm animal welfare standards as outlined in the WOAH Terrestrial Animal Health Code; (c) Implementing antimicrobial stewardship programs in animal agriculture*.”

To strengthen the operationalization of the One Health approach, we recommend adding to Article 5: “*5(5) The Parties shall establish a Joint One Health Coordination Mechanism, comprising representatives from WHO, WOAH, FAO, and UNEP, to oversee the implementation of One Health strategies under this Agreement. This mechanism shall: (a) Develop integrated surveillance systems for zoonotic diseases; (b) Coordinate cross-sectoral responses to emerging health threats; (c) Facilitate knowledge sharing and capacity building across human, animal, and environmental health sectors*.”

The treaty should include a new article specifically addressing animal health systems: “*Article 7bis. Animal Health Systems and Workforce: 1. The Parties recognize the critical role of animal health systems in preventing zoonotic diseases and shall commit to strengthening these systems as an integral part of pandemic prevention and preparedness. 2. Each Party shall: (a) Develop and maintain robust veterinary services, including adequate infrastructure and a skilled workforce; (b) Integrate animal health surveillance into national health information systems; (c) Ensure access to essential veterinary medicines and vaccines; (d) Promote the One Health approach in veterinary education and training*.”

Article 9 on research and development should be modified to explicitly include animal health research: “*9(3)The Parties shall promote and support research and development in animal health, including: (a) Development of vaccines for zoonotic diseases in animal reservoirs; (b) Improvement of diagnostic tools for early detection of zoonotic pathogens in animals; and (c) Research into sustainable animal management practices that reduce zoonotic disease risks*.”

In addition to the recommendations already outlined, we propose further revisions to ensure comprehensive coverage of animal welfare considerations. Article 10 on sustainable and geographically diversified production should be expanded to include animal health technologies. We recommend modifying Article 10(1) into: “The Parties commit to achieving more equitable geographical distribution and scaling up of the global production of pandemic-related health products, *including those for animal health*, and increasing sustainable, timely, fair and equitable access to such products, as well as reducing the potential gap between supply and demand during pandemics, through the transfer of relevant technology and know-how on mutually agreed terms.” Articles 11 and 12 on technology transfer and benefit-sharing should explicitly mention animal health technologies. We propose adding to Article 12: “*12(4) The WHO Pathogen Access and Benefit-Sharing System (PABS System) shall include provisions for sharing animal pathogen data and ensuring equitable access to animal health technologies developed from such data*.” Article 17 on whole-of-government and whole-of-society approaches should be modified to include animal welfare stakeholders. We suggest adding: “Each Party shall promote the effective and meaningful engagement of communities and other relevant stakeholders, including animal welfare organizations and veterinary professionals, as part of a whole-of-society approach in planning, decision-making, implementation, monitoring and evaluation.” Articles 19 and 20 on international cooperation and sustainable financing should explicitly mention support for animal health systems. We recommend adding to Article 20: “*20(6) The Coordinating Financial Mechanism shall allocate specific funding for strengthening animal health systems, improving animal welfare standards, and supporting One Health initiatives in developing country Parties*.”

### 6.2 Fostering ethical integration of animal welfare in global health policy

Beyond directly fulfilling key gaps in the treaty draft, the integration of animal welfare into the pandemic treaty that raises important ethical considerations must be carefully addressed to ensure effective and equitable implementation. We recommend that the treaty explicitly acknowledge the ethical dimensions of animal welfare in global health policy. It can be achieved by incorporating language that recognizes animal welfare as both an intrinsic value and a critical component of public health and pandemic prevention strategies. This approach aligns with the growing body of literature on the ethics of One Health, which emphasizes the interconnectedness of human, animal, and environmental wellbeing (210).

To address the challenges posed by varying cultural and economic contexts in implementing global animal welfare standards, we propose that the treaty adopt a principle of “progressive realization” of animal welfare standards. This approach, drawing inspiration from human rights treaties (211), would involve setting baseline welfare requirements that all signatories must meet, while encouraging and supporting nations to voluntarily adopt higher standards over time. Such a framework would allow for cultural sensitivity while still driving global progress in animal welfare. Furthermore, the treaty should promote ongoing ethical dialogue and research on the implications of animal welfare in global health policy. This could be facilitated through the establishment of an ethics advisory committee that would provide guidance on navigating complex ethical issues arising from the implementation of animal welfare provisions. The importance of such ethical considerations in global health governance has been highlighted by scholars in the field (212). By fostering a nuanced understanding of the ethical considerations at play, the treaty can help build consensus around the importance of animal welfare in pandemic prevention while respecting diverse cultural perspectives.

### 6.3 Strategies for effective implementation and capacity building

In order to ensure the implementation of the proposed animal welfare provisions, we recommend a multi-faceted approach focused on knowledge sharing, capacity building, and financial support. The treaty should establish a Global Animal Welfare and Health Network to facilitate the exchange of best practices, innovative technologies, and policy solutions among nations. This network would serve as a platform for collaborative learning and mutual support, helping countries overcome common obstacles in implementing animal welfare measures. Similar networks have proven effective in other areas of global health governance (213). Besides, we also propose the creation of regional centers of excellence for animal welfare and health. These centers would act as hubs for research, training, and policy development, tailored to the specific needs and contexts of different world regions. By localizing expertise and resources, these centers can play a crucial role in adapting global standards to regional conditions and providing targeted technical support to countries as they work to improve their animal welfare practices. After all, the success of regional approaches in advancing animal health and welfare has been demonstrated in various contexts (214). Further, the treaty should mandate the development of a comprehensive monitoring and evaluation framework. This framework should incorporate both quantitative metrics and qualitative assessments to provide a nuanced understanding of progress in implementing animal welfare provisions across different contexts. Regular reporting and review mechanisms should be established to track advancements, identify challenges, and share successful strategies among parties.

Lastly, recognizing the resource constraints facing many countries, particularly in the Global South, we recommend that the treaty include provisions for technical and financial assistance to support the implementation of animal welfare measures. Establishing a dedicated fund within the treaty's financial mechanism to support capacity building, infrastructure development, and technology transfer related to animal welfare and health should be considered. Equally important, partnerships with international financial institutions and development agencies should be further explored to leverage additional resources and expertise.

## 7 Conclusion

The COVID-19 pandemic has catalyzed an unprecedented global process to develop a new pandemic accord aimed at enhancing future prevention, preparedness, and response capacities. As this paper has demonstrated, incorporating animal welfare represents an essential, yet frequently overlooked, dimension for treaty effectiveness in reducing risks and protecting the wellbeing of humans, animals, and environments. The literature review highlights the growing body of evidence linking animal welfare, zoonotic disease prevention, and pandemic risks, underscoring the critical importance of integrating these considerations into the pandemic treaty. Through in-depth qualitative analysis of policy documents prepared by leading CSOs like FOUR PAWS, WCS, and A4AH between 2020 and 2023, including recommendations to the INB, policy briefs, responses to treaty drafts, and organizational reports, five major recurring themes emerged as priorities for pandemic policy: prevention of pathogen spillover, adoption of a One Health approach, strengthening animal health systems and infrastructure, improving sustainable and ethical animal management practices, and ensuring intersectoral policy coherence and governance. These themes demonstrate broad agreement among CSOs on what they “*want*” from the pandemic treaty. However, comparative analysis of the latest “Proposal for the WHO Pandemic Agreement” reveals significant divergences between existing draft provisions and CSO inputs on these vital areas.

To address these shortcomings, we have proposed detailed recommendations for revising and expanding key articles of the treaty. These recommendations aim to strengthen definitions, enhance guiding principles, and incorporate specific provisions for animal welfare and health throughout the treaty. We have also suggested strategies for ethical integration of animal welfare considerations and effective implementation of the proposed measures. The inclusion of robust animal welfare provisions in the pandemic treaty is not just ethically imperative but practically essential for safeguarding global health security. By addressing the animal-human-environment interface more comprehensively, the treaty can set a new standard for holistic, One Health-based approaches to pandemic prevention. Nevertheless, implementing these recommendations will require overcoming significant challenges, including varying cultural perspectives on animal welfare, resource constraints in low and middle-income countries, and potential resistance from industries affected by stricter animal welfare standards. Our proposed strategies for implementation and capacity building aim to address these challenges through knowledge sharing, regional cooperation, and targeted financial support.

The recent developments at the WHA, including the adoption of critical amendments to the IHR and the commitment to finalize the pandemic treaty by 2024 or 2025, underscore the timeliness and relevance of these research findings and recommendations. The momentum generated by these decisions provides a unique opportunity for governments and WHO decision-makers to consider and incorporate the insights and priorities of CSOs working at the intersection of animal welfare, conservation, and health into the ongoing treaty negotiations and implementation frameworks. As this paper has demonstrated, effectively preventing future pandemics necessitates dramatically expanding the scope of the pandemic accord to match scientific and civil society imperatives for transformative, integrated action. Governments can no longer afford to neglect or marginalize animal welfare as a peripheral concern divorced from human and ecological fates.

## Data Availability

The original contributions presented in the study are included in the article/supplementary material, further inquiries can be directed to the corresponding author.
